# Fast-conducting mechanonociceptors uniquely engage reflexive and affective pain circuitry to drive protective responses

**DOI:** 10.1101/2025.11.11.687663

**Published:** 2025-11-20

**Authors:** Karina Lezgiyeva, Jingyi Liu, Karen Nguyen, Michelle M. DeLisle, Frank C. Ko, Spencer Fullam, Alia M. Obeidat, Josef Turecek, Ilayda Alkislar, Brendan P. Lehnert, Rosa I. Martinez-Garcia, Riya Sivakumar, Jinheon Choi, Ofer Mazor, Lilit Garibyan, Nikhil Sharma, Alan J. Emanuel, Anne-Marie Malfait, Rachel E. Miller, David D. Ginty

**Affiliations:** 1 Department of Neurobiology, Harvard Medical School, 220 Longwood Avenue, Boston, MA 02115; 2 Howard Hughes Medical Institute, Harvard Medical School, 220 Longwood Avenue, Boston, MA 02115; 3 Department of Internal Medicine, Division of Rheumatology, Rush University Medical Center, Chicago, IL 60612, USA; 4 Wellman Center for Photomedicine, Department of Dermatology, Harvard Medical School, Massachusetts General Hospital, 50 Blossom St, Boston, MA 02114; 5 Department of Biochemistry and Molecular Biophysics, Vagelos College of Physicians and Surgeons, Columbia University Irving Medical Center, New York, NY 10032; 6 Department of Cell Biology, Emory University School of Medicine, 615 Michael Street, Atlanta, GA 30322; 7 Lead contact

## Abstract

Nociceptors detect damaging stimuli and evoke pain in healthy animals. We conducted an optogenetic activation screen to identify genetically defined nociceptor populations that elicit place aversion and nocifensive behaviors in response to stimulation. *Smr2*^*Cre*^- and *Bmpr1b*^*Cre*^-labeled Aδ high-threshold mechanoreceptors (HTMRs) emerged as two of the few nociceptor populations, and we focused on investigating their physiological, morphological, functional, and synaptic properties. These neurons densely innervate skin and other organs, are activated only by intense, potentially damaging stimuli, and are necessary for protective responses to sharp mechanical stimuli. Centrally, Aδ-HTMRs projections span multiple spinal segments and terminate across spinal cord laminae, forming strong, monosynaptic connections onto anterolateral tract projection neurons, including antenna cells of the deep dorsal horn. Aδ-HTMRs also engage a local spinal reflex circuit enabling a remarkably rapid limb withdrawal. Thus, Aδ-HTMRs are myelinated nociceptors with unique properties that can be exploited for development of new analgesics.

## Introduction

Despite its evolutionary importance and clinical burden, the biological underpinnings of pain remain incompletely understood. Pain is classified based on several features, including duration, anatomical location, and etiology^1,2^. Indeed, pain can be acute or chronic, somatic or visceral, and nociceptive, neuropathic, or nociplastic. Pain-causing stimuli range from potentially damaging mechanical, thermal, or chemical stimuli and inflammation to harmless or innocuous stimuli under certain pathological conditions. Mounting evidence suggests that different types of pain have different underlying mechanisms^3–10^.

Nociceptive pain, such as that caused by acute physical trauma or exposure to extreme temperatures, alerts us to harmful or damaging environmental stimuli and promotes avoidance. The neural signals underlying nociceptive pain originate in primary somatosensory neurons housed in dorsal root ganglia (DRG) or trigeminal ganglia and called nociceptors. Nociceptors can be categorized based on their soma size and conduction velocity as large, fast-conducting, myelinated A-fiber neurons and small, slow, unmyelinated C-fiber neurons^11,12^. Both A- and C-fiber nociceptors were first documented by Perl and colleagues in the cat in the 1960s^13,14^. Since then, nociceptors have been physiologically identified in every mammalian species studied, including mouse and human^11,15–21^. More recent RNA sequencing studies have confirmed the conservation of transcriptionally defined putative A- and C- fiber nociceptors across species^22–24^. The conduction velocity differences between the two main nociceptor types are consistent with clinical descriptions of “first pain” and “second pain”, and it has been suggested that the two stages of pain are driven by A- and C-fiber nociceptors, respectively^12^. In addition to latency differences, these two pain stages may be associated with distinct percepts and differentially susceptible to analgesics^25^. Yet, beyond their conduction velocity differences, the physiological differences between the two broad nociceptor classes and differences in the central circuits they engage to drive pain remain unclear.

Since pain is challenging to assess in non-human animals, definitions of nociceptors in preclinical research can be contentious. The neurons observed by Perl were categorized based on the original definition proposed by Sir Charles Sherrington in 1906^26^, which stated that a nociceptor is a neuron excited exclusively by noxious or damaging stimuli, and whose activation is accompanied by the perception of pain. This definition is often expanded to include sensory neurons with lower activation thresholds and wide dynamic ranges and whose responses scale from innocuous into the noxious range of stimuli^11^. Recent studies revealed that sensory neurons innervating hairy skin have mechanical thresholds that are not discrete but rather form a continuum and tile force stimulus space, making a clear distinction between low and high-threshold mechanoreceptors challenging^27,28^. Additionally, as advances in molecular biology have provided insight into immunohistological and transcriptional features associated with nociceptors, including expression of TrkA, CGRP, Substance P, TrpV1, Na_v_1.8, and IB4 binding, among others, molecular definitions are often used in place of physiological characterization^24,29^. Finally, there are challenges in distinguishing the properties of sensory neurons in healthy, uninjured animals versus animals in an altered state of disease, such as chronic pain or inflammation. The history of our understanding of *MrgprD*^+^ C-fiber neurons illustrates several of these points. These neurons were initially categorized as nociceptors based on their small soma size and IB4 binding^30,31^, without physiological or behavioral measurements. Further work revealed that *MrgprD*^+^ neurons can have mechanical thresholds similar to those of low-threshold mechanoreceptors (LTMRs), and their activation does not evoke a pain-like behavior in healthy mice^27,32,33^. However, in a model of neuropathic pain, activation of these neurons may drive a type of pain called mechanical allodynia^34^. To avoid ambiguity in describing sensory neuron functions, we propose a strict definition of nociceptors. Nociceptors are sensory neurons that respond to noxious or damaging stimuli and evoke pain and nocifensive behaviors in healthy, naive animals, whereas nociceptors and other sensory neuron types may contribute to nocifensive behaviors and the perception of pain in disease states such as neuropathic and inflammatory pain.

There are 15 or more distinct types of DRG sensory neurons, and mouse genetic tools now allow unprecedented access to most of these populations for observation and manipulation^27,28,35–48^. Over the preceding decade, most mouse genetics-enabled work has focused on small unmyelinated C fibers expressing TrpV1, Na_v_1.8 or CGRP, each of which label several somatosensory neuron types, along with some more specific populations such as those expressing *MrgprD*, *Sst*, and a few other molecular markers. Indeed, it is often suggested that most nociceptors are C-fiber neurons^12,37,42,43,49^. Comparatively few studies over the past decade have reported gaining genetic access to A-fiber high-threshold mechanoreceptors (A-HTMRs)^27,50,51^. Still, since their original discovery over 50 years ago, the morphologies, peripheral targets, functional properties, central projection patterns, and synaptic targets of A-fiber nociceptors remain poorly understood. Here, we leverage recently developed mouse genetic tools for a comparative behavioral analysis of most DRG sensory neuron types of the mouse to determine genetically defined A-fiber and C-fiber nociceptor populations. Two prominent transcriptionally distinct populations stand out as myelinated nociceptors and we undertook a detailed physiological, anatomical, morphological, synaptic, and behavioral characterization of these A-fiber nociceptors to understand their contributions to acute nociceptive pain.

## Results

### Two putative Aδ-HTMR populations drive uniquely fast and robust nocifensive behaviors and aversion

We first sought to identify the major nociceptor populations innervating glabrous skin of mice. For the purposes of this study, we define a nociceptor as a primary somatosensory neuron that fires action potentials in response to damaging or potentially damaging stimuli and, when activated, promotes both a nocifensive or protective behavioral response and an aversion to the source of the activating stimulus.

To identify sensory neuron subtypes that meet our behavioral definition of a nociceptor, we used mouse genetic tools for select sensory neuron subtype access and performed an optogenetic activation behavioral survey across 10 distinct cutaneous A-fiber and C-fiber DRG neuron subtypes. Most LTMR subtypes were excluded from this analysis because they are maximally responsive to innocuous stimuli^27^ and, when activated, they do not evoke nocifensive behaviors or, in the case of C-LTMRs and Aδ-LTMRs, they do not innervate glabrous skin^52^. The list of ten transcriptionally and physiologically defined cutaneous DRG neuron subtypes and the genetic tools used to label them are listed in Table 1.

**Figure 1.**
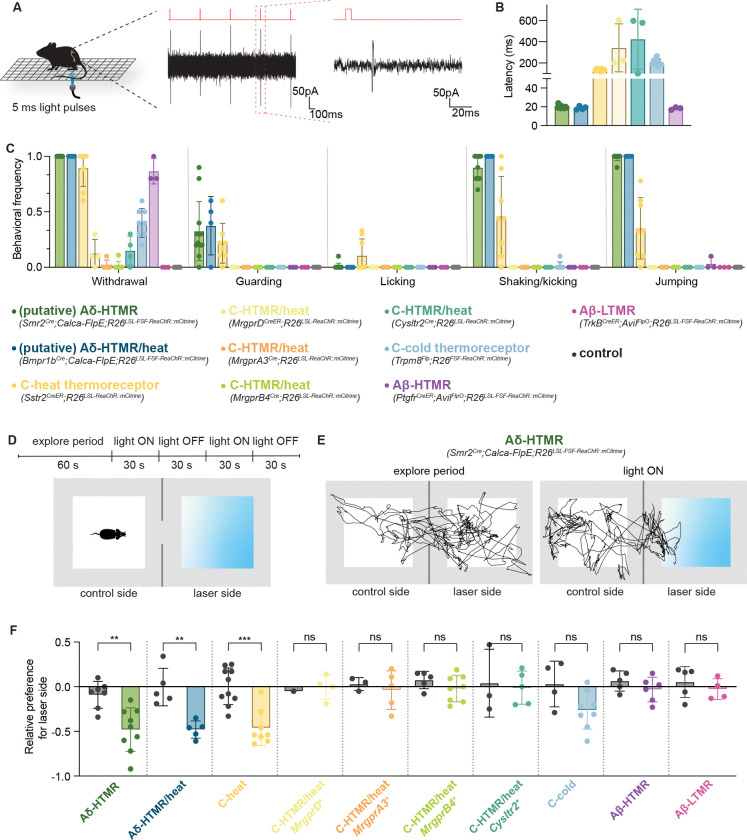
An optogenetic activation behavioral screen of DRG sensory neuron types (A) Left: Optogenetic stimulation paradigm. Animals were placed on a wire rack, and their hindpaw was illuminated with an LED from below. Right: *In vivo* loose patch electrophysiology reveals that 5-ms pulses trigger single action potentials in *Smr2*^*Cre*^-labeled neurons. Right trace is a zoomed-in version of the light-evoked spike outlined in red on the left trace. (B) Average latency (mean ± SD) of behavioral response to 5-ms light stimulation of glabrous hindpaw in mice expressing the opsin ReaChR in different sensory neuron populations. Each dot represents one animal (n = 12, 4, 8, 3, 3, 8, 3 from left to right). (C) Average frequency (mean ± SD) of select behavioral responses to 5-ms light stimulation of glabrous hindpaw in mice expressing ReaChR in different sensory neuron populations and littermate controls. See Supplemental Video 1 for a representative example of Aδ-HTMR-driven behavior. Littermate controls were animals that lacked either one of the driver alleles or the ReaChR allele. Each dot represents one animal (n = 12, 4, 8, 4, 5, 8, 4, 8, 3, 3, 3 from left to right). (D) Optogenetic real-time place preference paradigm. Animals were placed in a two-sided chamber in which the sides were separated by an arched doorway. The center of each chamber had transparent flooring, and a laser was placed beneath the floor on one side. (E) Representative locomotion traces from one animal expressing ReaChR in Aδ-HTMRs (*Smr2*^*Cre*^*; Calca-FlpE; R26*^*LSL-FSF-ReaChR::mCitrine*^) during the exploration (left) and the stimulation (right) periods. (F) Average relative preference (mean ± SD) for the laser side in mice expressing ReaChR in different sensory neuron populations and littermate controls. The relative preference for the laser side was calculated as the difference between fraction of time spent on the laser side during ON periods and fraction of time spent on the laser side during the exploration (laser off) period. Negative values indicate avoidance of the laser side and positive values indicate preference for the laser side. Littermate controls were animals that lacked either one of the driver alleles or the ReaChR allele. For *MrgprB4*^*Cre*^*; R26*^*LSL-ReaChR::mCitrine*^ animals, wild-type CD1 mice tested alongside the mutants were used as controls. Each dot represents one animal (n = 7, 9, 5, 5, 10, 8, 1, 4, 3, 5, 5, 8, 3, 5, 4, 8, 5, 6, 5, 4 from left to right, **p ≤ 0.01, ***p ≤ 0.001, unpaired t test).

To profile mouse behavioral responses to minimal activation of the different DRG sensory neuron subtypes, we optically stimulated the hindpaws of animals expressing the light-gated ion channel ReaChR in different sensory neuron populations and recorded their behavioral responses using high-speed videography (Figure 1A). Short 5-ms light pulses, which were shown to generate only a single action potential in *Smr2*^*Cre*^-labeled Aδ-HTMR neurons using *in vivo* electrophysiological recordings (Figure 1A), were delivered to the paws. Strikingly, when either the *Smr2*^*Cre*^-labeled or the *Bmpr1b*^*Cre*^-labeled A-HTMR populations were targeted, this stimulus was sufficient to reliably generate a rapid (~20 ms latency) and robust response characterized by paw withdrawal, shaking, and jumping, along with occasional guarding (Figure 1B–C, Supplemental Video 1). Activation of C-heat thermoreceptors also evoked a robust response, although with a much longer latency (200–400 ms). None of the four C-fiber polymodal sensory neuron subtypes produced a robust response upon activation. Likewise, as predicted, activation of light touch-responsive Aβ-LTMRs did not evoke an appreciable behavioral response. A lack of observable behavioral response was not due to a failure of opsin activation since light-evoked action potentials could be detected in the spinal cord upon stimulation of all neuron types that did not evoke a behavioral response^53^ (Figure S1). Activation of C-fiber cold thermoreceptors typically evoked a subtle limb withdrawal but no paw shaking, guarding, kicking, or jumping.

We also tested a mostly undescribed population of glabrous skin innervating neurons, which we refer to as Aβ-HTMRs. Aβ-HTMRs were first physiologically identified as moderate pressure receptors in 1968^15^ but have not been well characterized. This population of sensory neurons can be labeled with the *Ptgfr*^*CreER*^ mouse line and resembles the Aβ Field-LTMRs previously described in hairy skin^47^. The genetically labeled neurons have large soma diameters and express NFH but not CGRP (Figure S2A, B). Physiological recordings revealed that these neurons conduct action potentials in the Aβ velocity range and have indentation thresholds higher than those of Aβ-LTMRs but lower than Aδ-HTMRs (Figure S2C–F). Like other Aβ fiber neurons, Aβ-HTMRs have one central branch projecting directly to the dorsal column nuclei (DCN) and other branches that terminate densely in the LTMR recipient zone of the deep dorsal horn (Figure S2G, H). However, unique among other Aβ neurons, the peripheral axons of these large diameter CGRP-negative neurons penetrate the epidermis of the skin where they form free nerve endings (Figure S2I). Moreover, unlike the DCN-projecting Aβ-LTMR, the Aβ-HTMRs form expansive arbors in glabrous skin (Figure S2J, K). Interestingly, human glabrous skin also contains CGRP-negative epidermal endings that are NFH-positive in the dermis, and perhaps these correspond to mouse Aβ-HTMRs (Figure S2L). It is noteworthy that the mechanical response thresholds of these glabrous skin-innervating Aβ-HTMRs are comparable to those of *MrgprD*^*CreER*^-labeled C-HTMRs reported in hairy skin^27^. And, as observed with the four polymodal C-HTMR/heat subtypes, when Aβ-HTMRs were optogenetically activated, animals exhibited only a mild hindlimb withdrawal response, with no paw shaking, guarding, kicking, or jumping (Figure 1C).

To measure behavioral avoidance, we devised a real-time place preference paradigm. Animals expressing ReaChR in one of the ten sensory neuron subtypes were placed into a two-sided chamber with transparent floors, where one of the sides was illuminated from below with laser light (Figure 1D). We found that mice with ReaChR expressed in their Aδ-HTMRs or C-heat thermoreceptors, but not littermate controls, robustly and consistently avoided the laser side during illumination periods (Figure 1E, F). In contrast, stimulating any of the other seven sensory neurons did not lead to aversion to the stimulation side of the chamber (Figure 1F). This included Aβ-HTMRs, C-cold thermoreceptors, and the four polymodal C-fiber types, often referred to as nonpeptidergic nociceptors.

Thus, of the 10 cutaneous sensory neuron types tested, only three populations – two Aδ-HTMRs and C-heat thermoreceptors – meet our behavioral criteria of a nociceptor. While six of the neuronal populations evoked a modest paw withdrawal response in a subset of trials, they did not evoke paw shaking, guarding, kicking, or jumping responses, nor did they evoke place aversion. It is curious that none of the C-HTMRs met the nociceptor definition, as this suggests the possibility that acute mechanonociception is mediated exclusively by fast conducting neurons. Since the Aδ-HTMRs evoked the most rapid and robust responses in our pain behavioral measurements and because these neurons have been understudied, we focused our physiological, anatomical, and circuit-level analyses on these two Aδ-HTMR populations to gain insight into the somatosensory neuron basis of mechanical nociceptive pain.

### Neurons labeled by *Smr2*^Cre^ and *Bmpr1b*^*Cre*^ mouse lines are Aδ-HTMRs in mouse glabrous skin

We sought to define the physiological properties of genetically labeled A-fiber nociceptors in glabrous skin, where stimuli were delivered for the behavioral analyses, as most previous work on neurons with HTMR properties had focused on hairy skin^27,51^. Our findings revealed that while mechanical tuning properties of these genetically labeled neurons are consistent in hairy and glabrous skin, their responses to temperature differ depending on innervation target.

**Figure 2.**
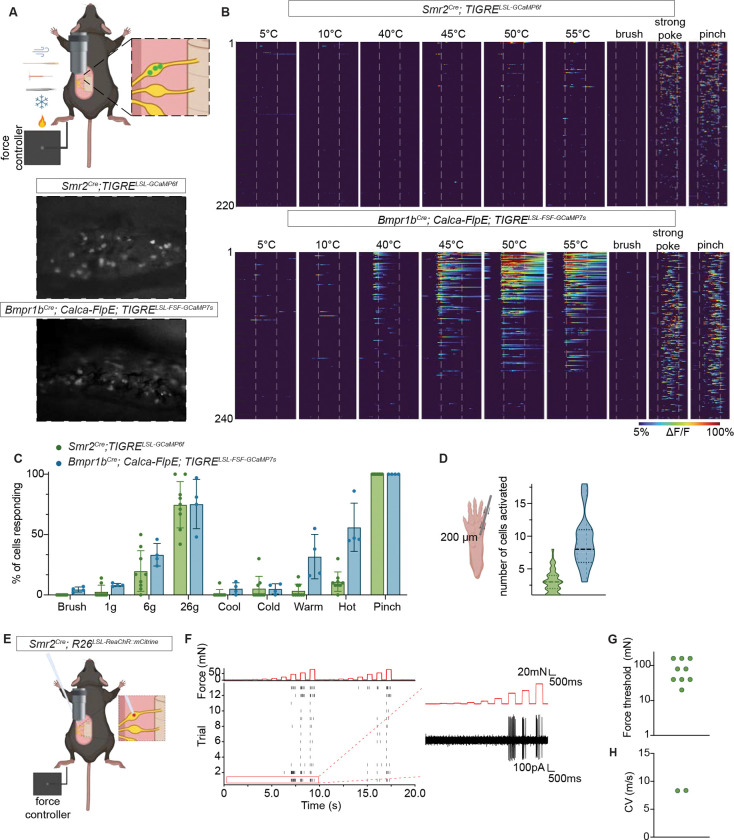
Physiological characterization of glabrous skin innervating neurons labeled by the *Smr2*^*Cre*^ and *Bmpr1b*^*Cre*^ mouse lines (A) *In vivo* epifluorescent calcium imaging. Stimuli were presented to the glabrous hindpaw, and calcium responses of genetically labeled sensory neurons were recorded in the cell bodies of lumbar level 4 DRGs. Representative fields of view are shown. (B) Calcium indicator responses of *Smr2*^*Cre*^- and *Bmpr1b*^*Cre*^-labeled neurons to select stimuli. Both neuronal populations responded to strong mechanical stimulation, but not to light brushing of the paw. Neither population responded to cold temperatures, and *Bmpr1b*^*Cre*^-labeled neurons had more pronounced responses to noxious heat compared to *Smr2*^*Cre*^-labeled neurons (n = 223 neurons across 9 animals for *Smr2*^*Cre*^-labeled neurons, 246 neurons across 3 animals for *Bmpr1b*^*Cre*^-labeled neurons). (C) Average percentage (mean ± SD) of neurons in a field of view responding to select stimuli. The number of neurons responding to pinch is used as a denominator. 1 g, 6 g, and 26 g are the weights of von Frey filaments. Cool is defined as 20 and 25°C, cold as 5, 10, and 15°C, warm as 35 and 40°C, and hot as 45, 50, and 55°C. Each dot represents an animal and an independent recording session (n = 9 for *Smr2*^*Cre*^-labeled neurons and 4 for *Bmpr1b*^*Cre*^-labeled neurons). (D) Number of cells in a field of view simultaneously responding to an indentation with a 200-μm probe tip (n = 84 indentation spots across 16 animals for *Smr2*^*Cre*^-labeled neurons, and 22 indentation spots across 4 animals for *Bmpr1b*^*Cre*^-labeled neurons). (E) *In vivo* targeted loose patch electrophysiological recordings. Stimuli were presented to the glabrous hindpaw, and somatic extracellular currents of genetically labeled sensory neurons were recorded. (F) An example raster plot of indentation responses of an *Smr2*^*Cre*^-labeled neuron. A current trace of the responses outlined with a red line is shown on the right. This neuron has a high indentation threshold, 40 mN, and fires repeatedly to ramp and hold mechanical stimuli. (G) Force thresholds of *Smr2*^*Cre*^-labeled neurons. These neurons have high mechanical thresholds, as all neurons recorded from required more than 10 mN of force provided with a 250-μm indenter to be activated. See Supplemental Figure 1 for a comparison between more broadly labeled sensory neuron populations. Each dot represents a unit recorded from a single animal (n = 9 units from 9 animals). (H) Conduction velocities of *Smr2*^*Cre*^-labeled neurons. The genetically labeled HTMRs exhibit an Aδ conduction velocity. Each dot represents a unit recorded from a single animal (n = 2 units from 2 animals).

We first used a high-throughput method of *in vivo* calcium imaging to observe and compare responses of *Smr2*^*Cre*^- and *Bmpr1b*^*Cre*^-labeled neurons to mechanical and thermal stimuli (Figure 2A). As previously observed in hairy skin^27,51^, both populations exhibited high mechanical force thresholds, showing no responses to brush and vigorous responses to higher forces, including skin indentation with stiff von Frey filaments, poking with closed forceps, and pinching (Figure 2B, C, Figure S3A). *Bmpr1b*^*Cre*^-labeled neurons have somewhat lower mechanical thresholds compared to *Smr2*^*Cre*^-labeled neurons (Figure 2C, Figure S3A). This difference was more pronounced when using von Frey filaments for stimulation compared to the 200-μm indenter tip (Figure S3A). This finding suggests that sharpness, in addition to force, may be a distinguishing feature for activating these two genetically defined Aδ-HTMR populations. We also observed that neurons of both populations have overlapping physiological receptive fields, as multiple neurons simultaneously responded to indentations delivered to a single spot with a 200-μm indenter tip (Figure 2D).

Consistent with the very high mechanical force thresholds of *Smr2*^*Cre*^-labeled neurons, we found that the mechanotransduction channel Piezo2 is dispensable for their responses (Figure S3B–D). This is in line with findings from both transcriptional and *in situ* hybridization analyses indicating that *Piezo2* expression is low in the *Smr2*^+^ population^35^ (Figure S3E) compared to other mechanosensory neuron types. Similarly, our lab’s previous work revealed that Piezo2 contributes to, but is not required for, mechanically evoked responses of *Bmpr1b*^*Cre*^-labeled neurons that innervate the colon^54^.

Interestingly, the thermal responses of the two glabrous skin innervating Aδ-HTMR subtypes were different from one another as well as their hairy skin counterparts (Figure S3F–G). In hairy skin, a minority of the *Bmpr1b*^*Cre*^-labeled neurons respond to noxious cold, and about half of the *Smr2*^*Cre*^-labeled neurons respond to noxious heat. In contrast, we found that in glabrous skin, few neurons of both populations exhibited appreciable cold responses, about half of the *Bmpr1b*^*Cre*^-labeled neurons responded to noxious heat, and *Smr2*^*Cre*^-labeled neurons showed minimal noxious heat responses (Figure 2B, C). Given the robust responses to heat in some of the *Bmpr1b*^*Cre*^-labeled neurons, the term Aδ-HTMR/heat is more appropriate for these neurons. The heterogeneous heat responses of glabrous skin-innervating *Bmpr1b*^*Cre*^-labeled neurons suggest the presence of cellular subtypes within this population, and their genetic identities, physiological features, and differential contributions to somatosensation remain to be investigated.

Prompted by the observation of skin type-dependent physiological differences in Aδ-HTMRs (Figure S3F–G), we also assessed the physiological responses of glabrous-innervating *Sstr2*^*CreER*^-labeled C-heat thermoreceptors using the *in vivo* calcium imaging platform. We observed that while some of the *Sstr2*^*CreER*^-labeled neurons responded to pinch, most were tuned to noxious heat, vigorously responding to 50–55°C (Figure S3H). Thus, both Aδ-HTMR subtypes and *Sstr2*^*CreER*^-labeled C-heat thermoreceptors respond almost exclusively to noxious, potentially damaging stimuli. These physiological properties align with the original definition of a nociceptor^13^ and, therefore, these three genetically labeled neurons can be classified as nociceptors based on both behavioral and physiological criteria.

We next used *in vivo* loose-patch electrophysiology to evaluate Aδ-HTMR action potential firing patterns, conduction velocities, and waveforms (Figure 2E–H). We focused on the *Smr2*^*Cre*^-labeled neurons because their response properties measured using calcium imaging were more homogenous. We confirmed the identity of the recorded neurons by shining light on the paw of mice expressing ReaChR in *Smr2*^*Cre*^-labeled neurons and observing spikes in the soma of targeted neurons. Using this method, *Smr2*^*Cre*^-labeled neurons were observed to produce action potentials with wide waveforms in response to high forces applied to the skin (Figure S3I). In addition, they fired repetitively and adapted slowly in response to ramp and hold mechanical stimuli (Figure 2F), although their firing patterns were much less stereotyped or reliable compared to those previously observed for Aβ-LTMRs^55^. Conduction velocity measurements of *Smr2*^+^ neurons (Figure 2H) confirmed that they fall within the Aδ range, similar to a previous report of hairy skin-innervating *Bmpr1b*^+^ neuron conduction velocity (Figure 2H; see^51^, neurons recorded there are likely *Bmpr1b*^+^; see also comparative conduction velocity measurements in Figure S2E – CGRP^+^ neurons recorded here are likely *Smr2*^+^ or *Bmpr1b*^+^ Aδ-HTMRs).

The homogeneity of physiological response properties of genetically labeled Aδ-HTMRs was reflected in their molecular and soma size homogeneity as determined by cell body immunolabeling and diameter measurements, respectively (Figure S4). Both *Bmpr1b*^*Cre*^- and *Smr2*^*Cre*^-labeled populations exhibit medium/large-diameter cell bodies, falling on the larger end of the CGRP^+^ cell body diameter spectrum (Figure S4C). Each population comprises ~45% of the medium/large diameter CGRP^+^ cells in lumbar DRGs (Figure S4D). *Smr2*^*Cre*^-labeled cells are relatively homogeneous in both cell diameter and molecular profile, while *Bmpr1b*^*Cre*^-labeled cells appear more varied. Nevertheless, virtually all Aδ-HTMRs of both types expressed CGRP and TrkA but did not bind IB4, a marker of non-peptidergic C-fiber neurons, or express Calb, a marker of A-fiber LTMRs (Figure S4E). The majority of genetically labeled Aδ-HTMRs also expressed NFH, consistent with their medium/large soma size (Figure S4). Expression of the heat-sensitive ion channel TrpV1 is interesting in light of our physiology data (Figure S4): most genetically labeled Aδ-HTMRs do not express detectable levels of TrpV1. The few neurons that do express TrpV1 may account for the hairy skin-innervating *Smr2*^*Cre*^-labeled neurons and the glabrous skin-innervating *Bmpr1b*^*Cre*^-labeled neurons that exhibited pronounced responses to heat (Figure 2B, C, Figure S3F, G). Neither Aδ-HTMR population expressed the ATP receptor P2XR3, suggesting that P2XR3-dependent purinergic signaling does not underlie sensory transduction in these neurons (Figure S4). Thus, *Smr2*^*Cre*^- and *Bmpr1b*^*Cre*^-labeled DRG neurons are transcriptionally, physiologically and molecularly distinct populations of Aδ-HTMRs, and together these two Aδ-HTMR populations account for most, if not all, medium/large diameter CGRP^+^, TrkA^+^ DRG neurons in the mouse.

### Aδ-HTMRs are required for normal responses to damaging mechanical stimuli

**Figure 3.**
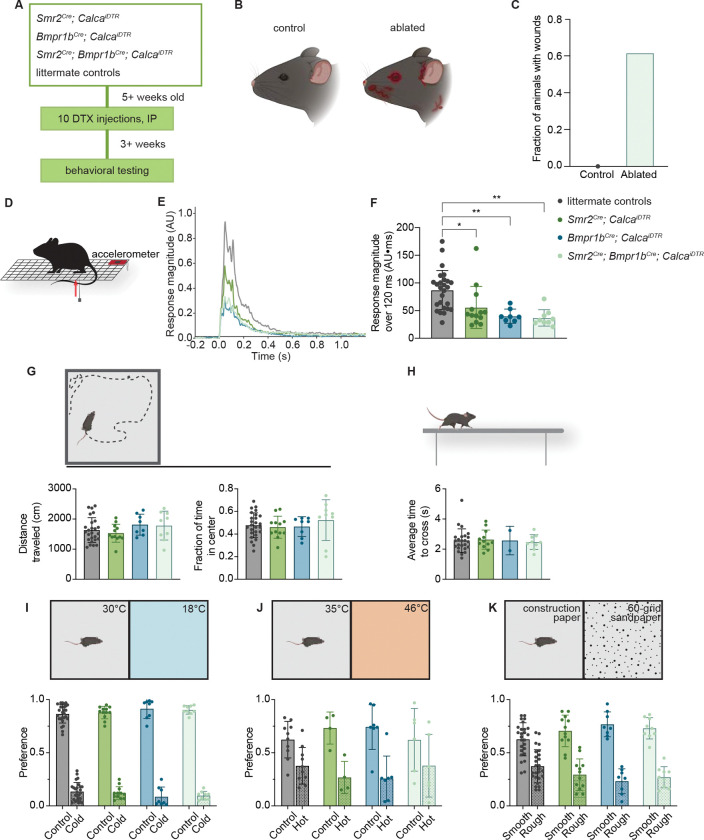
Aδ-HTMRs are required for behavioral responses to damaging mechanical stimuli (A) Schematic of the chemogenetic ablation paradigm. Adult mice expressing human diphtheria toxin receptor in one (*Smr2*^*Cre*^*; Calca*^*iDTR*^ or *Bmpr1b*^*Cre*^*; Calca*^*iDTR*^) or both (*Smr2*^*Cre*^*; Bmpr1b*^*Cre*^*; Calca*^*iDTR*^) Aδ-HTMR populations and littermate controls were treated with diphtheria toxin for 10 days. Behavioral testing was done three weeks after the last injection, or later. (B-C) Animals that had their Aδ-HTMRs ablated, but not littermate controls, developed facial and bodily wounds (0/12 controls and 16/26 ablated). (D) Pinprick behavior paradigm. Animals were placed on a wire rack, and a sharp pin was pushed into their hindpaws from below. Their movements were recorded using an accelerometer coupled to the wire rack. See Supplemental Video 2 for an example of the behavior. (E-F) Mice that had one or both of their Aδ-HTMR populations ablated exhibited diminished responses to pinprick stimulation. Animals that lacked either the *Cre* alleles or the *Calca*^*iDTR*^ allele and underwent toxin treatment were used as littermate controls. (E) shows average accelerometer responses from the different groups of mice. Each trace represents an average of multiple trials from several mice (n = 27 animals for controls, 14 for *Smr2*^*Cre*^*; Calca*^*iDTR*^, 8 for *Bmpr1b*^*Cre*^*; Calca*^*iDTR*^, 10 for *Smr2*^*Cre*^*; Bmpr1b*^*Cre*^*; Calca*^*iDTR*^). (F) shows response magnitude (mean ± SD) during the 120-ms period after stimulus onset. Each dot represents an animal (as in (E), n = 27, 14, 8, 10 from left to right; *p ≤ 0.05, **p ≤ 0.01, one-way ANOVA with Tukey’s multiple comparisons post-hoc test). (G) Chemogenetic ablation of Aδ-HTMRs does not affect animal locomotion, as evidenced by normal performance in the open field assay. The distance traveled (mean ± SD) and fraction of time spent in the center of the field (mean ± SD) are plotted. Each dot represents an animal (n = 26, 12, 8, 9 from left to right; no statistical difference between the groups, one-way ANOVA). (H) Chemogenetic ablation of Aδ-HTMRs does not affect animal locomotion and sensory-motor coordination, as evidenced by normal performance in the balance beam assay. Average times to cross the beam (mean ± SD) are plotted. Each dot represents an animal (n = 26, 12, 2, 6 from left to right; no statistical difference between the groups, one-way ANOVA). (I-K) Chemogenetic ablation of Aδ-HTMRs does not affect animals’ aversion to moderately cold (18°C) and hot (46°C) temperatures and rough texture. Preference is defined as the fraction of testing time spent on a given flooring, i.e., interacting with a given stimulus. Each dot represents an animal (for (I) and (K), n = 26, 12, 8, 9 from left to right; for (J), n = 10, 4, 7, 6 from left to right; no statistical difference between the groups, one-way ANOVA).

Since optical activation of either Aδ-HTMR population produced intense nocifensive responses and place aversion, we next asked whether one or both Aδ-HTMR subtypes are necessary for behavioral responses to noxious mechanical or thermal stimuli. To achieve Aδ-HTMR ablation, we used intersectional genetic strategies to generate mice expressing the human diphtheria toxin receptor in one or both populations and then delivered diphtheria toxin via intraperitoneal injections (Figure 3A). Strikingly, before any behavioral testing was performed, we observed that animals that had one or both Aδ-HTMR populations ablated developed facial and bodily wounds, while their co-housed littermate controls were unaffected (60% of Aδ-HTMR-ablated animals vs 0% of controls; Figure 3B–C, Figure S5A). One potential explanation for these wounds is excessive grooming or scratching. Alternatively, animals may fight more, or they don’t adequately protect themselves in fights with cage mates. This finding is reminiscent of tissue damage observed in human patients with loss-of-function pain disorders^56–59^ and suggests that a lack of fast mechanical pain signaling may lead to aberrant behaviors resulting in tissue damage.

We next asked whether Aδ-HTMRs are required for responses to noxious mechanical stimuli, as suggested by earlier work^50,60^. To do this, sharp pinpricks were delivered to animals’ hindpaws, and their movements were recorded using an accelerometer attached to the wire rack that they stood on (Figure 3D, E, Supplemental Video 2). These experiments were done using mice that had one or both Aδ-HTMR populations chemogenetically ablated and their littermate controls that lacked one or more alleles but that had received the toxin treatment. We found that ablating either or both Aδ-HTMR populations was sufficient to significantly reduce the animals’ responses to pinprick, despite incomplete ablation (Figure 3E, F, Figure S5B). Ablation of both populations led to a ~60% decrease in responsiveness.

This diminished response to pinprick in mice with Aδ-HTMR ablation was not due to a general motor deficit, as mutant and control mice performed similarly on both open-field and balance beam tests (Figure 3G, H). Animals’ aversion to moderate cold (18°C) and hot (46°C) temperatures was also unaffected by Aδ-HTMR ablation, consistent with our findings of a lack of physiological responses to cold and limited responses to heat (<45°C) in these populations (Figure 3I, J). All ablated groups also exhibited normal aversion to sandpaper flooring, suggesting that other DRG mechanosensory neuron types mediate rough texture aversion (Figure 3K). Together, these findings indicate that Aδ-HTMRs mediate normal protective responses to sharp and acute mechanical stimuli and are not necessary for responses to at least some other aversive stimuli.

### Two Aδ-HTMR populations densely innervate glabrous skin, forming a large overlapping meshwork of free nerve endings that penetrate the epidermis

To better understand how Aδ-HTMRs respond to noxious mechanical stimuli applied to glabrous skin, we next sought to describe their peripheral arborization and termination patterns. We observed that both Aδ-HTMR populations innervate glabrous skin of the paws densely and uniformly (Figure S6A).

**Figure 4.**
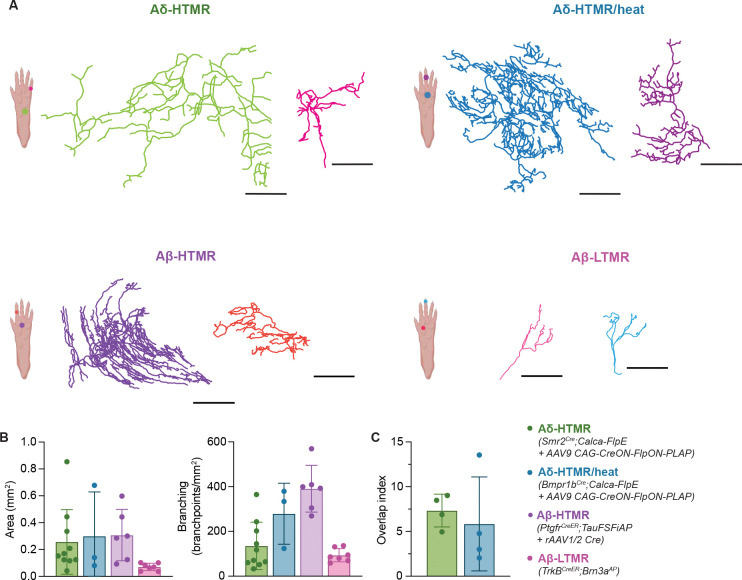
Aδ-HTMRs form expansive, highly overlapping terminal arbors in glabrous skin (A) Representative reconstructions of glabrous skin whole-mount AP staining of single Aδ-HTMRs (labeled with *Smr2*^*Cre*^ and *Bmpr1b*^*Cre*^), Aβ-HTMRs (labeled with *Ptgfr*^*CreER*^), and, for comparison, one Aβ-LTMR subtype (Aβ RA-LTMRs that form Meissner corpuscles, labeled with *TrkB*^*CreER*^). Details on sparse labeling strategies are provided in the Methods. For Aβ-LTMRs, the data shown were acquired from previous studies^27,88^ and re-analyzed and plotted. Reconstructions are color-coded based on the arbor location. Scale bars are 300 μm. (B) Area (mean ± SD) and branching (mean ± SD) of glabrous skin arbors of the four sensory neuron populations tested. Each dot represents a reconstructed arbor (n = 10, 3, 6, 7 from left to right). (C) Aδ-HTMRs have overlapping anatomical receptive fields. The average overlap index (mean ± SD) of the two populations is plotted. Each dot represents a single density comparison (see Methods for details; n = 4 pairs for both populations).

To visualize individual neurons of both Aδ-HTMR populations, sparse labeling experiments were done by injecting a low titer of CAG-CreON-FlpON-SEAP AAV9 virus into the glabrous hindpaw skin of *Smr2*^*Cre*^*; Calca-FlpE* and *Bmpr1b*^*Cre*^*; Calca-FlpE* animals (Figure 4A). This virus expresses alkaline phosphatase in a Cre- and Flp-recombinase-dependent manner. Consistent with our previous findings in hairy skin^27^, we observed that individual *Smr2*^*Cre*^- and *Bmpr1b*^*Cre*^-labeled neurons form large, expansive arbors in the skin, with the *Bmpr1b*^*Cre*^-labeled neurons branching more elaborately than *Smr2*^*Cre*^-labeled neurons but less so, by comparison, than *Ptgfr*^*CreER*^-labeled Aβ-HTMRs (Figure 4B). Aδ-HTMR arbors are much larger than those of Aβ-LTMRs and comparable in size to Aβ-HTMR arbors. Across the three A-fiber HTMR populations, axons terminating in the digits form smaller arbors compared to their palm-terminating counterparts. Inspired by the observation that 200 μm indentations activate multiple Aδ-HTMRs (Figure 2D), we also compared individual and population terminal densities to estimate the extent of homotypic overlap within a given area of skin. Consistent with the physiological measurements (Figure 2D), this morphological analysis revealed that 3–10 Aδ-HTMRs cover any given area of glabrous skin (Figure 4C, S6A). This is in contrast with the non-overlapping tiling pattern associated with LTMRs, including Aβ-LTMRs involved in fine discriminative touch^55,61^. This overlapping pattern of Aδ-HTMR skin innervation may provide better signal reliability at the expense of spatial acuity. Finally, when analyzing sectioned glabrous skin, both Aδ-HTMR populations were observed to penetrate the epidermis, where they lose their NFH-immunoreactivity (Figure S6B). This is distinct from the hairy skin-innervating *Bmpr1b*^*Cre*^-labeled neurons, which form circumferential endings around hair follicles in the dermis^27,51^. The epidermal endings of both Aδ-HTMR populations were devoid of S100^+^ surrounding lamellar cells, suggesting they are free nerve endings. Thus, as with the Aβ-HTMRs, which are not nociceptors by our criteria, the two Aδ-HTMR nociceptor subtypes densely innervate glabrous skin, where they penetrate the epidermis in a highly intermingled manner.

### Aδ-HTMRs innervate many tissues and organ systems of the body, including the musculoskeletal system, teeth, and internal organs

**Figure 5.**
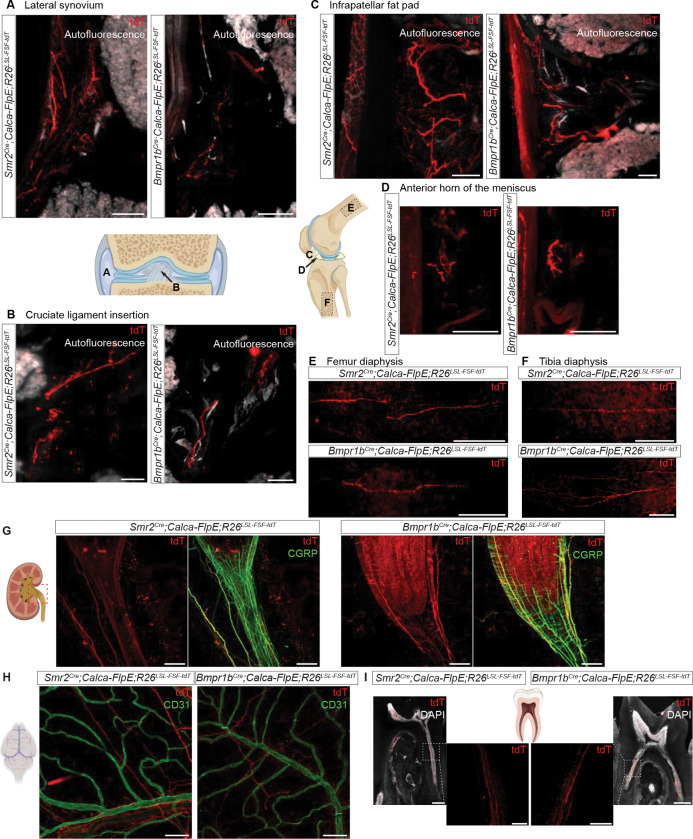
Aδ-HTMRs innervate many tissues and organ systems of the body (A-F) Representative light-sheet microscopy images from *Smr2*^*Cre*^*; Calca-FlpE; R26*^*LSL-FSF-tdT*^ and *Bmpr1b*^*Cre*^*; Calca-FlpE; R26*^*LSL-FSF-tdT*^ intact whole-mount-stained knee joints (A-D, 200-μm maximum intensity projections), femurs, and tibias (E-F, 100-μm maximum intensity projections) collected from 10-week-old mice (n = 2 animals/reporter line). tdTomato-labeled axons can be observed in: A) Lateral Synovium; B) Cruciate Ligament Insertion; C) Infrapatellar Fat Pad; D) Anterior Horn of the Meniscus; E) Femur Diaphysis; and F) Tibia Diaphysis. Scale bars are 200 μm. (G) Representative confocal images from *Smr2*^*Cre*^*; Calca-FlpE; R26*^*LSL-FSF-tdT*^ and *Bmpr1b*^*Cre*^*; Calca-FlpE; R26*^*LSL-FSF-tdT*^ whole-mount-stained kidneys. tdTomato-labeled axons are prominent in the renal pelvis and ureter regions. Scale bars are 200 μm. (H) Representative confocal images from *Smr2*^*Cre*^*; Calca-FlpE; R26*^*LSL-FSF-tdT*^ and *Bmpr1b*^*Cre*^*; Calca-FlpE; R26*^*LSL-FSF-tdT*^ whole-mount-stained cranial meninges. Blood vessels are labeled with CD31. tdTomato-labeled axons can be observed throughout the meninges. Scale bars are 100 μm. (I) Representative confocal images from *Smr2*^*Cre*^*; Calca-FlpE; R26*^*LSL-FSF-tdT*^ and *Bmpr1b*^*Cre*^*; Calca-FlpE; R26*^*LSL-FSF-tdT*^ molar sections. The reporter signal can be observed in the molar pulp. Scale bars are 200 μm for whole-tooth images and 50 μm for insets.

Inspired by observations of dramatic protective behaviors driven by skin-innervating Aδ-HTMRs, we next asked whether *Smr2*^*Cre*^- and *Bmpr1b*^*Cre*^-labeled neurons innervate other, non-cutaneous organs and tissues, especially those that may be associated with severe mechanically evoked pain. While future work will establish the physiological properties of *Smr2*^*Cre*^- and *Bmpr1b*^*Cre*^-labeled neurons found in organs and tissues other than the skin and colon, for the present study, we will refer to them as Aδ-HTMRs. We first examined bones and joints and found dense innervation by both Aδ-HTMR populations (Figure 5A–F). This is consistent with prior reports of CGRP^+^ Aδ-HTMRs in the bone^62,63^. In the knee joint, Aδ-HTMRs innervate the lateral synovium, the insertion of cruciate ligaments, infrapatellar fat pad (Hoffa’s fat pad), and the anterior horn of the meniscus (Figure 5A–D). Direct mechanical stimulation of these parts of the knee is associated with moderate to severe pain^64^, which may be mediated by Aδ-HTMRs.

Aδ-HTMR terminals of both types were also observed in the kidney and the walls of the urinary bladder (Figure 5G, Figure S6C), and previous work revealed innervation of the distal colon by the *Bmpr1b*^*Cre*^-labeled but not *Smr2*^*Cre*^-labeled neurons^54^. In the kidney, most Aδ-HTMR innervation was concentrated in the calyces, the renal pelvis, and the ureters. These kidney structures can experience increased mechanical pressure during inflammation or obstruction, for example, by kidney stones, and this increased pressure can cause severe pain.

Although our analyses focus on the Aδ-HTMRs found in the DRG and surveying the body, the trigeminal ganglia (TG) house mostly the same transcriptionally defined populations of somatosensory neurons that supply innervation of the head^28,35^. We confirmed that the neurons labeled by the *Smr2*^*Cre*^ and *Bmpr1b*^*Cre*^ lines in the TG are immunohistologically similar to those labeled in the DRG (medium/large diameter, CGRP^+^, IB^−^) (Figure S6D). In the head, we found that Aδ-HTMRs innervate both the meninges and tooth pulp (Figure 5H, I). Assuming these neuronal populations exhibit nociceptor properties like their glabrous skin-innervating counterparts, these findings suggest that they may contribute to tooth pain and headache pathogenesis, including migraine.

Thus, Aδ-HTMRs innervate a range of non-cutaneous tissues across the body, where they likely function as fast mechanonociceptors underlying nociceptive pain.

### Aδ-HTMRs terminate across multiple dorsal horn laminae and form nest-like structures in laminae III-IV

The unique physiological, morphological, and functional properties of the two Aδ-HTMR populations prompted a detailed characterization of their central connectivity patterns with the goal of defining central circuits that these nociceptors engage to mediate rapid nocifensive responses and pain perception. In the spinal cord, the termination patterns of Aδ-HTMRs were found to be highly distinct from other sensory neuron types. C-HTMRs and C-cold^27^ and heat thermoreceptor neurons terminate mainly in the superficial spinal cord dorsal horn, whereas Aβ-LTMRs^52,65,66^ and Aβ-HTMRs (Figure S2H) terminate in the deep dorsal horn.

**Figure 6.**
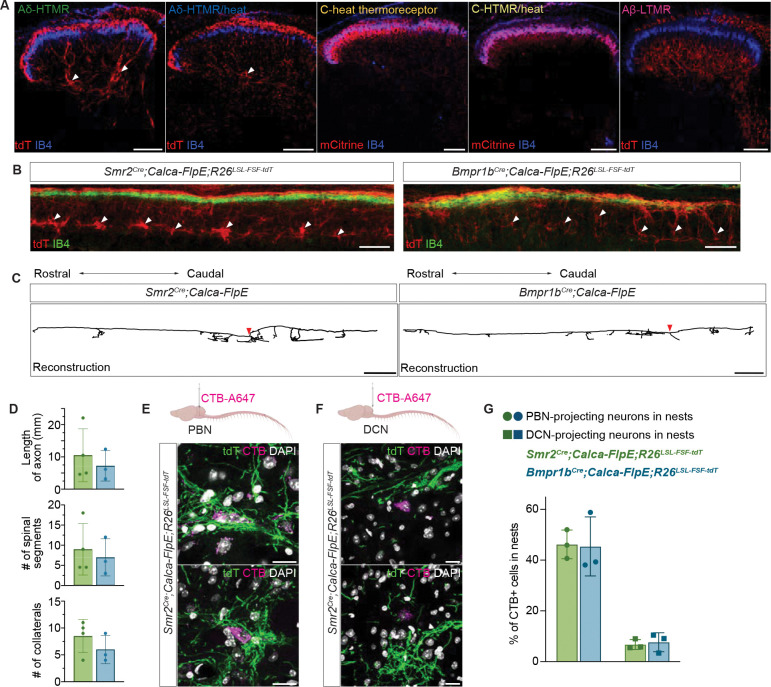
Aδ-HTMRs uniquely terminate across several laminae of the spinal cord dorsal horn and form collaterals that span multiple spinal segments (A) Aδ-HTMR spinal cord termination patterns are unique among DRG sensory neuron types. Representative confocal images of the two Aδ-HTMR (*Smr2*^*Cre*^*; Calca-FlpE; R26*^*LSL-FSF-tdT*^ and *Bmpr1b*^*Cre*^*; Calca-FlpE; R26*^*LSL-FSF-tdT*^), C-heat thermoreceptor (*Sstr2*^*CreER*^*; R26*^*LSL-ReaChR::mCitrine*^), C-HTMR/heat receptor (*MrgprD*^*CreER*^*; R26*^*LSL-ReaChR::mCitrine*^), and Aβ RA-LTMR (*TrkB*^*CreER*^*; Avil*^*FlpO*^*; R26*^*LSL-FSF-tdT*^) axons in the dorsal horn, taken from transverse sections of lumbar spinal cord. Arrowheads point at clusters of Aδ-HTMR axons in the deep dorsal horn. Scale bars are 100 μm. (B) Both Aδ-HTMR subtypes exhibit discontinuous terminals in the deep dorsal horn. Representative confocal images of *Smr2*^*Cre*^*; Calca-FlpE; R26*^*LSL-FSF-tdT*^ and *Bmpr1b*^*Cre*^*; Calca-FlpE; R26*^*LSL-FSF-tdT*^ spinal cord sagittal sections. Arrowheads point at clusters of Aδ-HTMR axons in the deep dorsal horn. Scale bars are 200 μm. (C) Aδ-HTMRs have long axons with many collaterals that span multiple segments of the spinal cord. Representative reconstructed axons of sparsely labeled Aδ-HTMRs. Red arrowheads indicate points of entry into the spinal cord. Scale bars are 1 mm. (D) Quantification of Aδ-HTMR central axon length along the rostrocaudal axis, number of spinal segments spanned, and number of collaterals (mean ± SD), related to examples shown in (C). Each dot represents an axon (n = 4 for *Smr2*^*Cre*^-labeled neurons and 3 for *Bmpr1b*^*Cre*^-labeled neurons). (E-F) Aδ-HTMRs form “nests” around antenna cell projection neurons of the anterolateral tract (ALT) (E) but not postsynaptic dorsal column neurons (PSDCs) (F). Representative confocal images from *Smr2*^*Cre*^*; Calca-FlpE; R26*^*LSL-FSF-tdT*^ and *Bmpr1b*^*Cre*^*; Calca-FlpE; R26*^*LSL-FSF-tdT*^ spinal cord sections. The projection neurons are retrogradely labeled using injections of the tracer CTB-A647 into the PBN (E) and DCN (F). Scale bars are 20 μm. (G) Percentage (mean ± SD) of CTB-labeled cells found within Aδ-HTMR nests. Quantification related to examples shown in (E-F). Each dot represents an animal (n = 3 for all groups).

Remarkably, unlike other sensory populations, Aδ-HTMRs terminate in both the superficial and deep laminae of the dorsal horn, forming clusters of axons in the latter (Figure 6A). In fact, when the two Aδ-HTMR populations were simultaneously labeled, most CGRP immunoreactivity in laminae III-V was observed to overlap with the Aδ-HTMRs (73%) (Figure S7A, C). Considering that the two Cre lines used for genetic labeling are <90% efficient^27^, we speculate that Aδ-HTMRs account for most, if not all, of the deep CGRP^+^ axon terminal immunolabeling. When both Aδ-HTMRs populations were ablated, albeit incompletely (Figure S5B), much of deep CGRP immunoreactivity was lost (Figure S7B, D), again supporting the idea that the two Aδ-HTMR subtypes provide most, if not all, of CGRP^+^ sensory endings in the deep dorsal horn. These genetic labeling experiments confirm and expand on earlier insights into Aδ-HTMR central anatomy achieved with single-axon reconstructions of physiologically defined neurons^21^. When viewed in sagittal sections, the termination patterns of Aδ-HTMRs are striking (Figure 6B). Superficially, in lamina I and II, a dense band of Aδ-HTMR terminals and fibers was observed. In contrast, in the deep dorsal horn, clusters of Aδ-HTMR terminals appeared discontinuous, potentially converging on sparsely distributed synaptic partners. This motif was especially pronounced in the *Smr2*^+^ population, perhaps reflecting distinguishing central connectivity patterns of this Aδ-HTMR population.

We also performed sparse genetic labeling experiments to determine the spinal cord innervation patterns of individual Aδ-HTMRs (Figure 6C). Unlike Aδ-LTMRs, C-LTMRs, and other C-fiber neuron types, which form a single terminally branched arbor in a rostrocaudally restricted manner^61,67^, individual Aδ-HTMRs produce multiple collateral arbors (5+) along the rostrocaudal axis, reminiscent of all Aβ-LTMR subtypes and proprioceptors^61^ (Figure 6C, D). This remarkable projection pattern likely enables Aδ-HTMRs to engage spinal cord circuit motifs across five or more spinal segments (Figure 6D). Additionally, axons of individual neurons were observed in both the superficial and deep lamina. This distinguishing feature of Aδ-HTMRs suggests that these neurons engage distinct postsynaptic partners along the dorsal-ventral axis of the spinal cord.

In both transverse and sagittal sections, we observed that the deep projections of Aδ-HTMRs were not diffuse and uniformly distributed, as has been observed with other sensory neuron subtypes, including Aβ-LTMRs^66^, but rather they appeared to envelop one or several cell bodies (Figure 6A, B), and this was especially apparent when counterstained with NeuN (Figure S7E). These striking anatomical structures, which we refer to as deep dorsal horn Aδ-HTMR “nests,” fascinated us as they may hold clues to the identity of central mediators of the unique behaviors evoked by Aδ-HTMRs. Therefore, we next sought to identify spinal cord neurons that reside within these deep dorsal horn Aδ-HTMR nests. There are several different neuronal populations in this laminar location, including two projection neuron types with large cell bodies – antenna neurons of the anterolateral tract (ALT) that project to the parabrachial nucleus (PBN) and other brain targets, and post-synaptic dorsal column neurons (PSDCs) that project to the dorsal column nuclei (DCN)^66,68^. To ask whether either or both projection neuron types are localized within the Aδ-HTMR nests, chrolera toxin B (CTB) retrograde tracer was injected bilaterally into either the PBN to label antenna cells or the DCN to label PSDCs (Figure 6E, F). We found that Aδ-HTMRs form nests that surround spinoparabrachial antenna neurons but not PSDCs (Figure 6G). Since antenna cells are sparse^68^ and Aδ-HTMRs nests often contain more than one cell body (Figure S7E), we suspect that these nests surround additional unknown neuronal populations. It is also noteworthy that the superficial Aδ-HTMR terminals reside in close proximity to lamina I spinoparabrachial neurons (Figure S7F).

### Aδ-HTMRs directly synapse onto distinct ALT projection neuron populations

**Figure 7.**
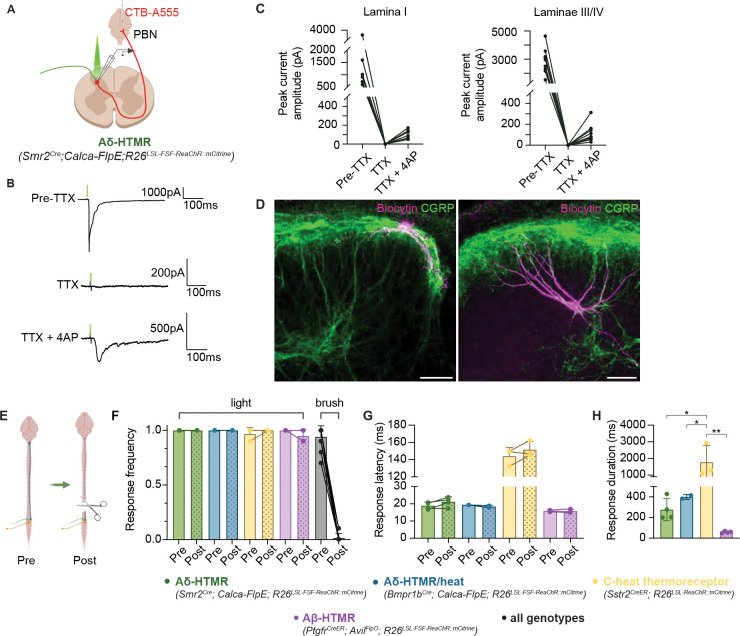
Aδ-HTMRs engage both a supraspinal circuit and a local reflexive circuit (A) Whole-cell patch clamp recordings of retrogradely labeled ALT projections neurons with optical stimulation of Aδ-HTMR axons in *Smr2*^*Cre*^*; Calca-FlpE; R26*^*LSL-FSF-ReaChR::mCitrine*^ spinal cord sections (300 μm). (B) Aδ-HTMRs form strong, monosynaptic connections onto ALT projection neurons. Representative traces of light-activated currents in a lamina III-IV ALT projection neuron; the response is abolished upon TTX administration and partially rescued with 4-AP, providing evidence for a monosynaptic connection. (C) Peak current amplitude of light-activated currents in superficial (lamina I) and deep (laminae III-IV) ALT projection neurons. Each dot represents a recording from one cell (6 cells across 4 animals for superficial cells, and n = 9 cells across 4 animals for deep cells). Importantly, of 9 deep cells recorded, all exhibited substantial light-evoked EPSCs that persisted upon TTX + 4-AP application. On the other hand, of 8 superficial cells recorded, 2 exhibitted only weak light-evoked EPSCs; the latter two cells are not plotted. The average before-drug-application evoked currents are ~2.9 nA for deep (lamina III/IV) spinoparabrachial neurons and ~1.4 nA for superficial (lamina I) spinoparabrachial neurons. (D) Representative images of biocytin-filled superficial (left) and deep antenna (right) spinoparabrachial neurons. Cells were filled at the end of the recordings summarized in (A-C). Scale bars are 50 μm. (E) Schematic of spinal transection experiment. The spinal cord was cut above spinal segment T9, and mice were allowed to recover for 6 hours before testing. (F) Light-evoked activation of Aδ-HTMRs promoted hindpaw withdrawal in spinalized animals, while brush-evoked responses were lost. 5-ms light pulses and brush stimuli were delivered to the hindpaws of *Smr2*^*Cre*^*; Calca-FlpE; R26*^*LSL-FSF-ReaChR::mCitrine*^, *Bmpr1b*^*Cre*^*; Calca-FlpE; R26*^*LSL-FSF-ReaChR::mCitrine*^, *Sstr2*^*CreER*^*; R26*^*LSL-ReaChR::mCitrine*^, and *Ptgfr*^*CreER*^*; Avil*^*FlpO*^*; R26*^*LSL-FSF-ReaChR::mCitrine*^ animals before (Pre) and after (Post) spinalization. See Supplemental Videos 3 and 4 for representative examples of Aδ-HTMR and C-heat thermoreceptor-driven behaviors after spinalization. Each dot represents an animal (n = 4, 2, 4, 12 from left to right). (G) Spinal transection does not alter the light-evoked response latency. Each dot represents an animal, the same as (F). (H) Optical activation of physiologically distinct sensory neuron types evokes reflexive responses of varying duration. See Supplemental Video 5 for an example of prolonged behavioral response to activation of C-heat thermoreceptors. The assay was performed in spinalized animals. Each dot represents an animal (n = 4, 2, 3, 4 from left to right, *p ≤ 0.05, **p ≤ 0.01, one-way ANOVA with Tukey’s multiple comparisons post-hoc test).

Because of the close proximity of Aδ-HTMR terminals to both superficial and deep ALT projection neurons, we hypothesized that Aδ-HTMRs form monosynaptic connections upon them. To test this idea, we performed whole-cell patch clamp electrophysiological recordings of retrogradely labeled ALT neurons in spinal cord slices while optically activating ReaChR-expressing *Smr2*^*Cre*^-labeled Aδ-HTMRs (Figure 7A, B). These experiments revealed that 100% (nine out of nine) of recorded retrogradely labeled antenna cells within the deep dorsal horn received strong monosynaptic inputs from *Smr2*^*Cre*^-labeled Aδ-HTMRs (Figure 7C). Post hoc histological analysis of the biocytin-filled antenna cells revealed CGRP signal around both the cell body and the dorsally directed dendrites (Figure 7D), suggesting multiple loci for Aδ-HTMRs synaptic input. In the superficial dorsal horn, six out of eight recorded spinoparabrachial neurons received Aδ-HTMR input which proved to be monosynaptic (Figure 7C). This heterogeneity in the superficial spinoparabrachial neuron responses may reflect the presence of distinct ALT populations in the superficial dorsal horn^69–72^, as prior work showed that *Nk1r*^+^ but not the *Gpr83*^+^ ALT projection neurons receive direct, monosynaptic input from CGRP^+^ sensory neurons^73^. The striking differences in the location and morphology of the superficial and deep dorsal horn ALT projection neurons (Figure 7D) raises questions about whether these neurons might differentially process inputs from Aδ-HTMRs and other afferents. Overall, these findings show that Aδ-HTMRs have highly unique central projections that form strong monosynaptic connections onto both superficial and deep dorsal horn ALT projection neurons that extend to the PBN and likely other target regions of the brain.

### Aδ-HTMRs are the primary drivers of an ultrafast, spinally-mediated limb withdrawal reflex

Motivated by the observation that optical activation of ReaChR expressing Aδ-HTMRs evokes invariable limb withdrawal with a short, 20–25 msec latency, we next asked whether this response is mediated by a local spinal reflex. For this, the spinal cord of animals expressing ReaChR in their Aδ-HTMRs was transected at thoracic segment 9 (T9), leaving a potential local lumbar spinal reflex intact (Figure 7E). A complete loss of motor responses to brushing the hindpaws was observed in the spinalized animals. However, when light pulses were applied to the paw, a fast withdrawal and paw shaking behavior was consistently observed, although, unsurprisingly, the response did not involve whole-body movement as compared to control mice (Figure 7F, G, Supplemental Video 3). This finding indicates that Aδ-HTMR activation is sufficient to drive short-latency limb withdrawal and paw shaking through a spinal reflex. We also observed that activating *Sstr2*^*CreER*^-labeled C-heat thermoreceptors and *Ptgfr*^*CreER*^-labeled Aβ-HTMRs in spinalized animals evoked a reflexive paw movement (Figure 7F, G). Interestingly, the movement pattern, magnitude, and duration differed from those evoked by Aδ-HTMR activation, suggesting that these physiologically distinct sensory neurons may engage distinct spinal reflex motor circuits (Figure 7H, Supplemental Videos 4, 5). Importantly, although Aβ-HTMRs evoke a modest reflexive paw withdrawal when optically activated, these neurons are not classified as nociceptors based on either a behavioral or a physiological definition, underscoring the notion that fast reflexes cannot be equated to nociception or pain and are mediated by distinct circuits. Thus, Aδ-HTMRs engage both fast local spinal circuits and slower ascending pathways to drive nocifensive behaviors and aversion.

## DISCUSSION

We conducted a optogenetic activation screen of somatosensory neuron types in naive healthy mice to identify genetically labeled nociceptor populations based on a physiological and behavioral definition rooted in early work of Sherrington^26^ and Perl^13^. Two Aδ-HTMR populations and a C-heat thermoreceptor population emerged from our analyses as nociceptors. While it is possible that a 5-ms light pulse was not an optimal stimulus for all studied sensory neuron populations, it was sufficient to evoke electrophysiological responses in the spinal cord, and the differential responses to this identical stimulus might reflect different baseline levels of nociceptive circuitry engagement by the afferents. It is also revealing that the three nociceptor populations identified in our screen had mechanical and thermal thresholds that were so high that continued application of such stimuli would result in tissue damage^27^, consistent with Perl’s original definition. Importantly, our findings highlight that other commonly cited sensory neuron features are poor predictors of nociceptor identity, including cell body diameter, broad molecular markers such as Na_v_1.8 expression or IB4 binding, and an “HTMR” designation. Additionally, it is worth emphasizing that the small number of nociceptor populations identified in our screen is a result of our rather strict nociceptor definition. We do not argue that these are the only sensory neurons capable of driving pain. Instead, we propose that the term “nociceptor” is most useful when studying nociceptive pain in healthy animals. Indeed, activation of LTMRs and other sensory neuron types may evoke nocifensive reflexes and the perception of pain after nerve injury, upon inflammation, and in other disease states^34,74,75^.

Here, we characterized and compared three distinct A-fiber HTMR populations, two of which are nociceptors. Importantly, while mouse Aδ-HTMRs are nociceptors and Aβ-HTMRs are not, this conduction velocity distinction is likely to be less predictive of function in humans. Indeed, A fiber HTMRs are generally faster in humans than in mice and there is evidence that a human nociceptor population corresponding to mouse hairy skin-innervating *Bmpr1b*^+^ Aδ-HTMRs conducts at Aβ conduction velocity^18,22,76^. In the mouse, we find that the three genetically labeled A-fiber HTMRs have distinct but overlapping physiological and morphological properties – relatively high mechanical thresholds and expansive peripheral arbors that penetrate the epidermis. However, their central anatomy and, presumably, synaptic partners are distinct. Like the Aβ-LTMRs, Aβ-HTMRs have diffuse projections that terminate within the LTMR recipient zone of the deep dorsal horn and a branch projecting to the DCN. Aδ-HTMRs, on the other hand, terminate within both the superficial and deep dorsal horn where they form “nests” around and synapse onto the ALT projection neurons, and they do not have DCN-projecting axons. These findings emphasize the significance of afferents’ central connectivity to their function.

Aδ-HTMRs drive a remarkably fast local spinal reflex, and establishing genetic access to the first-order neurons in this nociceptive withdrawal reflex circuit can now enable identifying the cellular components of this long-recognized but unresolved circuit^26,77^. Aδ-HTMRs also provide strong monosynaptic inputs to ALT projection neurons across dorsal horn laminae. The role of the ALT in pain processing is well established, although most work in the spinal cord has focused on the ALT projection neurons of the superficial dorsal horn^19,70,71,73,78,79^. The antenna cells of the deep dorsal horn appear to have distinct intrinsic electrophysiological properties^80^, and they are poised to receive different synaptic inputs compared to their superficial counterparts based on their laminar location. Indeed, the Aδ-HTMRs are unique in forming nests around antenna cell bodies, presumably enabling their remarkably strong synaptic connections. How Aδ-HTMR signals are integrated with other sensory inputs to the superficial and deep dorsal horn to shape ALT projection neuron firing patterns is an exciting area of future research.

Aδ-HTMRs densely innervate skin and other organs. While further analysis of the properties of these neurons in non-cutaneous tissues is warranted, in long bones^62^, kidneys^81^, bladder^82^, cranial meninges^83^, and teeth^84^, Aδ fibers, and Aδ-HTMRs in particular, have been electrophysiologically identified, making it likely that, at least in those tissues, the *Smr2*^*Cre*^- and *Bmpr1b*^*Cre*^-labeled neurons have physiological properties and functions similar to their cutaneous counterparts. Indeed, in the distal colon, *Bmpr1b*^*Cre*^-labeled neurons function as Aδ-HTMRs^54^. Across organs, Aδ-HTMRs may contribute both to normal tissue homeostasis and protection and to excessive and maladaptive pain in disease. In osteoarthritis, for example, Na_v_1.8^+^, CGRP^+^ nerve fibers sprout in the knee joint and subchondral bone and become sensitized^85–87^. A genetic handle on the two Aδ-HTMR populations will allow for a deeper understanding of the role these neurons play in both health and disease.

Given the role of Aδ-HTMRs in acute nociceptive pain and the potential for mouse genetics-enabled discovery of their role in other pain states, access to the full transcriptional profile of these neurons across species may yield great benefits for therapeutic development through identification of potentially druggable targets. Finding Aδ-HTMR-specific transducers and activity modulators may enable targeted control of these neurons. For instance, since Aδ-HTMRs can be mechanically activated by a Piezo2-independent mechanism, genetically labeled Aδ-HTMRs are an excellent model for identifying molecular mediators of noxious force-evoked mechanotransduction. Establishing these mediators may not only expand our understanding of mechanosensation but also prompt development of inhibitors to attenuate excessive mechanonociception and pain.

In conclusion, work presented here establishes two genetically labeled Aδ-HTMRs as myelinated nociceptors with unique physiological, morphological, and synaptic features distinguishing them from C-fiber nociceptors and Aβ-HTMRs. These findings open avenues for advancing our understanding of the neurobiological underpinnings of pain in health and disease and the development of new analgesics.

## Methods

### Mice

All mice used in this study were maintained on a mixed background. Adult mice (1.5–20 months old) were used, unless specified otherwise. Both male and female mice were used for all experiments. Mice were handled and housed in accordance with Harvard Medical School and IACUC guidelines. Mice were kept in a temperature- and humidity-controlled room with a 12-hour light/dark cycle, with food and water available *ad libitum*.

*Smr2*^*Cre*^ (strain #039562, ^27^), *Bmpr1b*^*Cre*^ (strain #039561, ^35^), *Sstr2*^*CreER*^ (strain #039563, ^27^), *MrgprD*^*CreER*^ (strain #031286, ^37^), *MrgprB4*^*Cre*^ (strain #021077, ^39^), *Cysltr2*^*Cre*^ (strain #039985, ^27^), *Trpm8*^*FlpO*^ (strain #039564, ^27^), *TrkB*^*CreER*^ (strain #027214, ^38^), *R26*^*LSL-FSF-tdTomato*^ (strain #021875, ^90^), *R26*^*LSL-FSF-ReaChR::mCitrine*^ (strain #024846), *R26*^*LSL-ReaChR::mCitrine*^ (strain #026294), *R26*^*LSL-tdTomato*^ (strain #007914, ^91^), *TIGRE*^*LSL-GCaMP6f*^ (strain #030328, ^92^), *TIGRE*^*LSL-FSF-GCaMP7s*^ (strain #034112), *Tau*^*FSFiAP*^ (strain #039981, ^93^) mice are available at Jackson Laboratory. The following mouse lines have been previously described: *MrgprA3*^*Cre* 40^, *Calca-FlpE* ^73^, *Avil*^*FlpO* 73^, *Calca*^*iDTR* 94^. The *Ptgfr*^*CreER*^ mouse line was generated at the Janelia Research Campus Gene Targeting and Transgenic Facility using CRISPR-based homologous recombination techniques in embryonic stem (ES) cells by inserting a CreER cassette into the first coding exon of the *Ptgfr* gene. Chimeras were produced via blastocyst injection, and germline transmission was confirmed through standard tissue genotyping PCR. The *R26*^*FSF-ReaChR*^ line was derived from the *R26*^*LSL-FSF-ReaChR::mCitrine*^ line by germline excision of LSL cassette.

### Tamoxifen treatment

Tamoxifen (Sigma, T5648) was dissolved in sunflower oil and stored at −80°C until used. Tamoxifen was delivered intraperitoneally (i.p.). *Sstr2*^*CreER*^ animals were treated with 2 mg at P21; *MrgprD*^*CreER*^ animals were treated with 2 mg at P21; *Ptgfr*^*CreER*^ animals were treated with 1 mg at P21; *TrkB*^*CreER*^ animals were treated with 0.5 mg at P5. At least 3 weeks were allowed for Cre induction before using mice for experiments.

### DTX treatment

Diphtheria toxin (Sigma, D0564) (DTX) was dissolved in saline and stored at −20°C until used. DTX was administered i.p. over 10 days at a dose of 20 ng/g of mouse weight per day. Ablation efficiency was quantified with *in situ* hybridization (RNAScope).

### Optical activation behavior

#### Setup

Mice were habituated to the room and holding chambers for 2 days before the experiment. On the day of the testing, mice were placed on top of a wire rack in holding chambers for 10 minutes to acclimate. 470 mN LED (Thorlabs, M470F3) was used with Ø1000 μm, 0.39 NA optic fiber (Thorlabs, M35L01) and 532 nm, f=7.86 mm, NA=0.51 collimator (Thorlabs, F240SMA-532) for optical stimulation. LED power was measured to be ~130 mW/cm^2^. 5-ms light pulses were triggered using a custom MATLAB and Arduino program and delivered from below to the plantar surface of animal hindpaws. Each animal received 12 stimuli to alternating hindpaws, each stimulus 5 seconds apart. Mouse behavior was recorded with a high-speed camera (Edmund Optics, Cat. #11–506) at 200 fps.

#### Analysis

Videos of mouse behavior were viewed and analyzed manually. Stimuli were observed on the video, and the latencies to select behaviors were calculated by counting frames elapsed. The first 10 stimulus presentations were considered to calculate behavior frequency. The assay and quantification were performed blind to genotypes.

### Real-time place preference

#### Setup

One week prior to testing, animals were moved to the testing room and habituated to investigator handling by undergoing tail inking. Animals were group habituated to the black matte test chamber (12 in L × 6 in W × 7 in H), center divided with an arched doorway and allowed to freely explore for 5 minutes. The test chamber was mounted on an optically clear acrylic floor with an opaque 1-in border along the chamber walls on each side of the chamber to create a safety zone for escape from painful laser stimulation. A laser and three LED lights (Thorlabs, M470L5 #M01048055; LEDD1B driver #M01063661) were mounted below the floor, oriented to illuminate up through the floor of the stimulation side of the chamber. The LEDs were mounted 1/2 inch below the floor surface to create a 3-in halo of light stimulation overlapping each LED to fill the entire floor with light. To encourage travel to the light stimulation side, the illumination power was graded from 7–10 mW/cm^2^ closest to the door opening to 10–12.5 mW/cm^2^ in the center of the floor to 14–15.5 mW/cm^2^ along the far wall. During the room/chamber habituation, animals were also habituated to the LED light source at very low (~0 mW) power. The room and test platform were lined with odorless 1-in thick soundproof panels (Bonded Logic Natural Fiber Acoustic Sound Absorbing panels, Model #60600–11212).

The test lasted 3 minutes and consisted of 5 stages.

Stage 1: 1 minute - exploration period. Mice were allowed to explore both sides of the chamber with no illumination.

Stage 2: 30 seconds - Light ON. The “laser” side of the chamber was illuminated.

Stage 3: 30 seconds - Light OFF. No illumination.

Stage 4: 30 seconds - Light ON. The “laser” side of the chamber was illuminated.

Stage 5: 30 seconds - Light OFF. No illumination.

Following test completion, the animal was removed from the testing chamber and returned to their home cage. The equipment was cleaned, and the system was reset for the next animal.

A custom Bonsai script was used to trigger the LEDs and lasers and Imaging source USB 2.0 camera. Mouse locomotion was recorded with a top-down camera at 60 fps.

#### Analysis

Animal locomotion was tracked, and time spent on each side was calculated with a custom MATLAB program. The assay and quantification were performed blind to genotypes.

### Pin prick assay

#### Setup

Mice were habituated to the room and holding chambers for 2 days before the experiment. Importantly, when the same animals were tested in any other assay, the pin-prick assay was done last. The same setup was used as for the optical activation behavior, with modifications. A house-made pinprick apparatus was added; upward movement of a sharp pin (FST, 26007–02) was triggered with a manual push-button. An accelerometer (SparkFun, ADXL335) was secured to the wire rack on which the mice were standing to pick up their movements. To ensure that data consist only of trials where the pin made strong contact with the paw skin, a circuit was set up to measure conductance between the pin and the mouse paw that rested on a conductive metal grid. A DAQ board (National Instruments, USB-6002) was used to synchronously record accelerometer signals along with button presses and skin contact signals. These data were collected for 3 minutes as an experimenter delivered multiple pinpricks to the plantar surface of animals’ hindpaws. Animal behavior was also recorded with a camera at 200 fps.

#### Analysis

Custom MATLAB code was used to read and analyze recorded analog data. Each push of a button was considered a putative trial. Data from all trials were visually inspected. The criteria for selecting a trial for further analysis included 1) pin-to-skin contact initiated after the start of the trial, 2) conductance value for pin-to-skin contact value exceeding 1.5 V, which was subjectively deemed to correspond to strong contact when analyzing corresponding videos, and 3) low baseline accelerometer values (movement) before the start of the trial. Stimulus onset was determined to be at the time of pin to skin contact. Movement onset was empirically determined to be at the time when accelerometer value reached 0.15 V. For trials that passed the quality control, the movement magnitude was computed as area under the curve for accelerometer values across 120 ms after stimulus onset. For presentation, accelerometer voltage readings were converted to arbitrary units (AU) such that 1 AU = maximum accelerometer voltage reading across all trials. The assay and quantification were performed blind to genotypes.

### General methods for open field, balance beam, temperature preference, and texture preference assays

The assays were performed as previously described^95^. The assays were run in the following order: open field, balance beam, temperature preference (cold), temperature preference (hot), texture aversion, pin prick. Mice were provided with chew snacks and always habituated to the testing room at least 30 minutes before handling. Animals were briefly group-habituated to the test chambers. The chambers were cleaned with ECOS unscented dish soap and 70% ethanol between animals. All assays and quantifications were performed blind to genotypes.

### Open field assay

The testing chamber was made of white acrylic (10 in L × 8 in W × 8 in H). Construction paper (Pacon^®^ Tru-Ray) was placed on the floor of the chamber. After habituation, the mice were placed in the testing chamber alone for 5 minutes. Their activity was recorded with a top-down camera at 30 fps. A custom MATLAB program was used to track locomotion and calculate time traveled and time spent in the center of the chamber.

### Temperature preference assay

The testing chamber used was made of black acrylic (12 in L × 6 in W × 7 in H) with an arched doorway across the center and rested on 2 metal temperature control plates (TE Technology, TC-720). For habituation, both plates were set to 30°C. For cold temperature testing, one plate was set to 30°C (control) and the other to 18°C (cold). For hot temperature testing, one plate was set to 35°C (control) and the other to 46°C (hot). After habituation, the mice were placed in the testing chamber alone for 5 minutes. Their activity was recorded with a top-down camera at 30 fps. A custom MATLAB program was used to track locomotion and calculate time spent on either side of the chamber.

### Texture aversion assay

The testing chamber used was made of black acrylic (12 in L × 6 in W × 7 in H) with no divider and rested on an acrylic floor. For habituation, construction paper (Tru-Ray Heavyweight, Pacon) was placed on the floor. For testing, half of the floor was covered with construction paper, and the other half was covered with coarse 60-grit sandpaper (ceramic version, 26060PGP-4, 3M). After habituation, the mice were placed in the testing chamber alone for 10 minutes. Their activity was recorded with a top-down camera at 30 fps. A custom MATLAB program was used to track locomotion and calculate time spent on either side of the chamber.

### Balance beam assay

Animals were trained to walk on the beam for 2 days prior to testing. The balance beam was constructed of 1 m matte white acrylic with a flat 12 mm surface. The beam was mounted 50 cm above the bench top. A soft pad was stretched below the beam to serve as a soft-landing surface to cushion falls. A black matte acrylic box was placed at each end of the beam to provide a start and end enclosure for the animal. During the training, the animals were placed on the beam and encouraged to move across it towards an enclosure. On testing day, animals were placed into the enclosure on one end and allowed to cross. Four beam crossings were recorded with top-down and side cameras at 60 fps. Videos were manually analyzed to determine time to cross, number of slips, and number of stalls.

### Reflex assay

#### Transection surgery

Spinal transection was performed as previously described^96^. Mice were anesthetized with inhaled isoflurane and injected with carprofen at a dose of 5 mg per kg of mouse weight. Ophthalmic ointment was used to protect the eyes. The fur below the neck was shaved and removed with Nair. 100 μL of 2% lidocaine (Covertus, 2468) was injected subcutaneously around the incision. A skin incision was made over the thoracic segment of the spinal column, and the vertebral muscles were removed to expose the gap between spinal segments T9 and T10. Spring scissors were then inserted into the gap, and the spinal cord was cut. To ensure a complete cut, a curved blade was then inserted into the gap and moved laterally. Care was taken to avoid cuts to the underlying lungs. The incision was then sutured, and the mouse was allowed to recover for 6 hours before testing. A transection was deemed successful if lower limb paralysis was observed. Mice were euthanized after the testing.

#### Setup

The same general procedure as for the optogenetic activation behavior was used. The mice were tested the day before the spinal transection surgery and 6 hours after the transection. After the transection, the animals’ lower limbs were paralyzed, so the hindpaws were moved down through the wire rack for better stimulus access. Brush, light, and pinch stimuli were applied to the paws of the animals. Animal behavior was recorded with a high-speed camera at 200 fps. Only the animals with reduced responses to brushing and retained responses to pinching were considered for analysis.

#### Analysis

Videos of mouse behavior were analyzed manually, similar to optogenetic activation behavior. Frames were counted from stimulus onset to movement onset (latency) and from movement onset to movement conclusion (duration).

### Skin injections

Mice aged P7–10 were anesthetized with inhaled isoflurane. AAV9-CAG-FLEX-hPLAP (1.19E+13 gc/mL, custom-made by Janelia Viral Core) or AAV2/9-CAG-CreON-FlpON-SEAP (9.18E+13 gc/mL, custom-made by Boston Children’s Hospital Viral Core) virus was injected into the glabrous skin of hindpaws in a volume of 1.5–2 μL with a borosilicate glass pipette at various dilutions (1:100 to 1:150) for sparse labeling. To label glabrous innervating DRG neurons for loose patch electrophysiology, 2 μL of 2 μg/mL CTB-A555 (Fisher, C34776) was injected into the glabrous skin. A small amount of Fast Green dye was mixed into the virus or CTB solution to visualize the injection. Care was taken to avoid blood vessels and potential systemic spread of the virus.

### Perfusion

Mice were anesthetized with a high dose of inhaled isoflurane and transcardially perfused with ~10 mL of 1X PBS and ~15 mL of 4% PFA in 1X PBS. Unless specified otherwise, tissues were collected and post-fixed in 4% PFA rotating at 4°C overnight, then washed with 1X PBS 3 times for ~10 minutes to avoid overfixation.

### Glabrous skin collection

This tissue collection protocol was used for immunohistochemistry but not whole-mount alkaline phosphatase staining. Mice were deeply anesthetized with ketamine (125 μg/g) and xylazine (12.5 μg/g). Glabrous skin was dissected off the paw while the mouse was anesthetized and placed in 1% PFA at 4°C for 2 hours. The skin samples were then washed in 1X PBS and microdissected further as described below. The samples were immediately processed. The mouse was euthanized or perfused, if other tissues were collected.

### Whole-mount alkaline phosphatase (AP) staining

The staining was performed as previously described^93^. 3 weeks after virus injection, mice were perfused and tissues were collected as described. Post-fixed and washed paws and spinal columns were used for these experiments. Glabrous skin was removed from the hindpaw in one piece, and any remaining fat, connective tissue, and hypodermis were cleaned off. Spinal cords were microdissected from spinal columns with DRG attached. All microdissected tissues were moved to fresh 1X PBS and incubated for 2 (skin) or 2.5 (spinal cord) hours at 67°C. Tissues were then washed in AP detection buffer (0.1 M Tris pH 9.5, 0.1 M NaCl, 50 mM MgCl_2_, 0.1% Tween-20) 3 times for 5 minutes at room temperature. Substrate was then provided (3.4 μL of NBT and BCIP in AP detection buffer), and the tissues were incubated in the dark with slight agitation for approximately 24 hours. To stop the reaction, tissues were then pinned flat on a sylgard plate to prevent curling and incubated in 4% PFA at room temperature for 1 hour. Finally, tissues were dehydrated with serial incubation in 50%, 70%, and 100% ethanol at room temperature for 1 hour each and stored in 100% ethanol until imaging at 4°C. Spinal cords were incubated in 100% methanol for a few hours prior to imaging. When ready, tissues were incubated in BABB (1 part Benzyl Alcohol, 2 parts Benzyl Benzoate) until clear and imaged in BABB under Zeiss AxioZoom stereoscope in brightfield mode.

### Single neuron morphological quantifications

Images were acquired as described above of single neurons in the skin and spinal cord for which a clear single axon could be traced out of the tissue. Images were viewed and analyzed in ImageJ with the SNT plugin. Individual peripheral and central arbors were reconstructed using SNT. For peripheral arbors, branchpoints were manually counted, and area was calculated by drawing a tight polygon surrounding the arbor. For central arbors, collaterals were manually counted, and the number of spinal segments was approximated based on the number of spinal roots.

### Overlap index quantification

Images from sparsely labeled (as described above) and densely labeled (from *Smr2*^*Cre*^*; Calca-Flp; Tau*^*FSFiAP*^ and *Bmpr1b*^*Cre*^*; Calca-Flp; Tau*^*FSFiAP*^ mice) skin were compared for this analysis. A square of defined area was drawn around similar hindpaw regions on two images. Then, all axons in the field of view were reconstructed, the reconstructed images were binarized, and black and white pixels were counted. Axon density was defined as the number of black pixels divided by the total number of pixels in the field of view. Axon density in the sparsely labeled image was divided by the axon density in the densely labeled image to get an overlap index value.

### Cryosectioning

In preparation for cryosectioning, microdissected tissues were placed in 30% sucrose at 4°C for 1–3 nights or until the tissue sank. Tissues were then placed in cryomolds in Optimal Cutting Temperature (OCT; Andwin Scientific Tissue-Tek^™^ CRYO-OCT Compound) compound and frozen over dry ice. Samples were sectioned at 20 μm (DRG) or 30 μm (all other tissue types) directly onto Superfrost Plus slides and dried at room temperature in the dark overnight.

Teeth within the mandibles were decalcified prior to the sucrose cryoprotection step in Morse’s solution (22.5% formic acid, 10% tri-sodium citrate in Milli-Q water) for 30–40 hours with rotation at room temperature as previously described^97^.

### Immunohistochemistry

All imaging was performed with an LSM-900 confocal microscope, unless otherwise specified.

#### Staining skin, kidney, and teeth samples (cryosections)

ImmEdge Pen was used to draw a border around the tissue on the slide. Slides were rehydrated for 5 minutes with 1X PBS and then washed 2 times for 5 minutes with 0.1% TritonX-100 in 1X PBS. The slides were then incubated in blocking solution (5% normal donkey serum, 0.1% TritonX-100 in 1X PBS) for 2 hours at room temperature. Primary antibodies were added in blocking solution, and slides were incubated overnight at 4°C. After washing with 0.1% TritonX-100 in 1X PBS, secondary antibodies were added in blocking solution, and the slides were incubated for 2 hours at room temperature. The slides were then washed again and mounted with DAPI Fluoromount (SouthernBiotech, 0100–20).

#### Staining DRG and spinal cord samples (cryosections)

ImmEdge Pen was used to draw a border around the tissue on the slide. Slides were washed with 1X PBS 3 times for 5 minutes and then incubated in blocking solution (5% normal donkey serum, 0.1% TritonX-100 in 1X PBS) for 2 hours at room temperature. Primary antibodies were then added in blocking solution and slides were incubated at 4°C overnight. Slides were washed 4 times for 5 minutes with 0.02% Tween-20 in 1X PBS. Secondary antibodies were added in blocking solution, and the slides were incubated for 2 hours at room temperature. Slides were washed again and mounted with DAPI Fluoromount (SouthernBiotech, 0100–20).

#### Staining sagittal spinal cord sections (free-floating)

The staining was performed in a 12-well plate. 60 μm cryosections were collected directly into the wells containing 1X PBS. All steps were performed with gentle agitation. Sections were incubated in blocking solution (5% normal donkey serum, 0.3% TritonX-1200 in 1X PBS) for 1 hour at room temperature and then transferred to blocking solution containing primary antibodies overnight at 4°C. After 3 washes with 1X PBS, sections were incubated in blocking solution containing secondary antibodies for 2 hours at room temperature. After 3 more washes, sections were mounted on a slide with DAPI Fluoromount (SouthernBiotech, 0100–20) using a paintbrush.

#### Staining kidney and bladder samples (whole-mount)

Post-fixed and washed tissues were used. Kidneys were bisected lengthwise to improve antibody penetration. Tissues were washed with 1% TritonX-100 in 1X PBS for 6–8 hours at room temperature, changing solution every 30 minutes. Tissues were then transferred into blocking solution (5% normal donkey serum, 20% DMSO, 1% TritonX-100 in 1X PBS) with primary antibodies and incubated for 3 nights with rotation at room temperature protected from light. After another 6–8 hours of washes, tissues were incubated in blocking solution with secondary antibodies for 3 nights with rotation at room temperature protected from light. The tissues were then washed for 6–8 hours again, dehydrated with serial incubation in 50%, 70%, and 100% methanol at room temperature for 1 hour each, and stored in 100% methanol. The tissues were then cleared by incubating in BABB. An imaging well was made on a tissue slide with vacuum grease, and a coverslip was placed on top. The bladder was cut to flatten.

#### Staining cranial meninges (whole-mount)

Skullcaps were post-fixed and washed as described. Meninges were carefully peeled off the skull. The meninges were incubated in blocking solution (20% normal donkey serum, 0.3% TritonX-100 in 1X PBS) for 1 hour at room temperature. Primary antibodies were then added to the blocking solution, and the tissue was incubated overnight at 4°C. After the tissue was washed with 0.3% TritonX-100 in 1X PBS 3 times for 1 hour at room temperature, secondary antibodies were added to the blocking solution, and the tissue was incubated overnight at 4°C. The tissues were washed again, then rinsed with 1X PBS 2 times and carefully mounted on slides with Fluoromount Gold. A paintbrush was used to flatten the meninges.

### Joint whole-mount immunolabeling and lightsheet microscopy imaging

Knee joints collected from male and female naïve mice aged 10 weeks (n = 2 knees/reporter line) were fixed and decalcified in Morse’s solution for 3 days. Decalcified knee joints were then processed for immunolabeling and tissue clearing as previously described^97,98^. In brief, decalcified knee joints were dehydrated and rehydrated with methanol gradient and decolorized with hydrogen peroxide. The samples were then immunolabeled with RFP antibody (Rockland, 600–401-379, 1:1000) for 2 weeks, followed by a secondary antibody conjugated with AF647 fluorophore (Thermo Fisher Scientific, A21245, 1:1000) to boost the RFP signal for 2 weeks. Immunolabeled samples were embedded in 1% agarose with the patellar tendon facing upwards. After agarose embedding, samples were subsequently dehydrated in methanol gradient, delipidated in dichloromethane, and cleared in dibenzyl ether. Cleared knee joints were imaged by ZEISS Lightsheet 7 (ZEISS) to visualize RFP positive nerves in 3D (Zen Blue 3.7). An air gap between the refractive index matching solution and objective was corrected by setting the objective correction collar at 1.56.

### Human skin immunohistochemistry

#### Biopsy

IRB-approved study was conducted at the Massachusetts General Hospital (Boston) between March 2021 and April 2021 to obtain skin biopsies from 4 healthy, adult subjects. Written, informed consent was obtained before participation.

Skin biopsies (3 mm) were obtained from distal palmar surface of 4^th^ or 5^th^ digits, processed, and analyzed according to consensus standards^99^. Tissue was placed in Zamboni’s fixative (Newcomer Supply) for 3 hours at room temperature, then washed in 1X PBS 3 times.

#### Immunohistochemistry

Glabrous skin samples were cryoprotected in 15% sucrose in 1X PBS overnight at 4°C, then transferred to 30% sucrose in 1X PBS overnight at 4°C. Tissue was embedded in OCT (1437365, Fisher), frozen using dry ice, and immediately cryosectioned (20 μm) and collected onto glass slides. Sections were allowed to dry overnight at 4°C. Slides not used for staining immediately were placed at −20°C. To reduce background, sections were first rehydrated in 1X PBS, washed once in 0.1% Triton X-100 in 1X PBS for 15 minutes, followed by 3 5-minute washes with 1X PBS, then incubated in Image-iT^™^ FX Signal Enhancer (ThermoFisher, I36933) for 45 minutes at room temperature. Sections were then washed 3 times for 5 minutes in 1X PBS and incubated in blocking solution (5% normal donkey serum, 0.1% TritonX-100 in 1X PBS) for 2 hours at room temperature. Slides were incubated with primary antibodies diluted in blocking solutions at 4°C overnight, washed 3 times for 20 minutes each with 0.1% TritonX-100 in 1X PBS, and then once for 5 minutes with 1X PBS. A second short block was performed for 30 minutes before being incubated with secondary antibodies diluted in blocking solutions for 1.5 hours at room temperature. Slides were then washed again 5 times for 10 minutes each with 0.1% TritonX-100 in 1X PBS, and then once for 5 minutes with 1X PBS. Finally, slides were mounted in DAPI Fluoromount (SouthernBiotech, 0100–20).

### DRG image quantification

Images were analyzed in ImageJ. Maximum intensity projections were obtained, and cells with clearly visible DAPI nuclei were analyzed. Ellipses were drawn around cells of interest, and area was measured; cell diameter was calculated from the area. The diameters for each cell type were then sorted, and values below the 20^th^ percentile were discarded to minimize the chance of including cell fragments. For Supplementary Figure 3P, large diameter was defined as >25 μm.

### Spinal cord image quantifications

For reporter and CGRP overlap quantifications, signals in the two channels were independently thresholded using the triangle method. Area occupied by pixels with above-threshold values was calculated and used for the overlap calculation.

For calculating the CGRP intensity in control and HTMR-ablated animals, sections from control and “ablated” animals were collected onto the same slides to minimize experimental variation. All sections were imaged using the same settings. Images were rotated such that most of the IB4 signal was horizontal. Superficial dorsal horn was defined to be above the IB4 band; deep dorsal horn was defined to be up 250 μm below the center of the IB4 band. Rectangles were drawn around the superficial and deep dorsal horn, and mean gray value in the CGRP channel was measured. Mean gray values were normalized to the highest mean gray value obtained from images on the same slide.

### *In situ* hybridization

Mice were deeply anesthetized with inhaled isoflurane, decapitated, and their spinal columns were extracted and placed on ice. Fine DRG microdissection was performed in dissection medium (DMEM:F12 (1:1) supplemented with 1% pen/strep and 12.5mM D-Glucose) atop ice. DRG were immediately placed in OCT compound in cryomolds and frozen in dry ice-cooled 2-methylbutane. Samples were cryosectioned at 20 μm thickness and stored at −80°C until further use. *In situ* hybridization was done using the ACDBio RNAScope platform (RNAscope^™^ H202 and Protease Reagents, RNAscope^™^ Multiplex Fluorescent Detection Kit v2, RNAscope^™^ probes, and Akoya Opal dyes). The following probes were used: *Bmpr1b* (Cat. # 533941), *Smr2* (Cat. # 538931), *Calca* (Cat. # 420361), *Piezo2* (Cat. # 1228811), *GCaMP* (Cat. # 557091).

To quantify ablation efficiency, cells expressing one or several transcripts of interest were counted in ImageJ. Only cells with a visible DAPI nucleus were counted. For the *Calca* channel, only cells with bright signal were counted. For the *Smr2* and *Bmpr1b* channels, cells with more than 3 bright puncta were counted. Quantifications were done blind to genotypes.

### Retrograde labeling of spinal cord projection neurons

Mice were anesthetized with inhaled isoflurane and placed in a small animal stereotaxic frame (David Kopf Instruments). Sustained-release buprenorphine (0.1 μg/g) was injected subcutaneously before starting the surgery. Ophthalmic ointment was applied to the eyes, and body temperature was maintained around 37°C with a heating pad. A freshly thawed aliquot of 10 μg/mL CTB-A647 (Fisher, C34778) was used for injections. A small amount of Fast Green dye was mixed into the CTB to visualize the injection. A borosilicate glass pipette and a Microinject system (World Precision Instruments) were used for the injections. Mice were used for experiments 3 days after the injections.

#### DCN injections

DCN were exposed as previously described^93^. In a stereotaxic frame, the animal’s neck was bent 45°. Paraspinal muscles were retracted to expose the gap between the base of the skull and the C1 vertebra. The dura was nicked with a bent insulin syringe. A pipette was lowered 150–200 μm below the brain surface near the obex. 75 nL of CTB was injected in 2 sites bilaterally (4 injections total) at a rate of 50 nL/min.

For labeling DCN-projecting sensory neurons, rAAV1/2-Cre virus (Penn Viral Core, 2E+12 gc/mL) or AAV2-retro-hSyn-FlpO virus (Boston Children’s Hospital Viral Core, 2.06E+14 gc/mL) was injected into the DCN of P11 mice.

#### PBN injections

Injection was performed as previously described^73^. Skull surface was leveled, burr holes were drilled with a dental drill, and 150 nL of CTB was injected in 2 sites bilaterally (4 injections total) at a rate of 75 nL/min. For more reliable anterior-posterior axis PBN targeting, a correction factor F was used (F = (bregma - lambda)/4.21). The following coordinates were used: x = ±1.3–1.4, y = 5.0–5.2*F, z = 2.85–3.10 from the brain surface.

### Calcium imaging

#### Setup

The imaging and stimulus delivery were performed as previously described^27^. Mice were anesthetized with inhaled isoflurane, and their body temperature was maintained at 37°C throughout the experiment. The back hair was shaved off, and Nair was applied to remove the remaining hair. An incision was made over the lumbar spine and paravertebral muscles overlaying vertebrae L3-L5 were loosened and pulled away from the bone. The spine was stabilized at the L3-L4 vertebrae with a custom-made clamp. The bone covering L4 DRG was removed with Friedman-Pearson Rongeurs (FST, 16221–14). Bleeding was controlled with Surgifoam (Mckesson, Cat. # 1972) and cotton swabs. The right thigh hair was shaved. The right hindpaw was secured on the imaging platform, glabrous side up, with silicone putty.

The surgical preparation was then transferred to the platform under an upright epifluorescence microscope (Zeiss Axio Examiner) with 10X air objective (Zeiss Epiplan, NA = 0.20). 470 nm LED (Thorlabs, M470L5) with LED driver (Thorlabs, LEDD1B) was used as the light source. A CMOS Camera (Thorlabs, CS505MU1) was triggered at 10 fps with a 50 ms exposure time. All the recorded stimuli were synchronized with the camera and LED using a DAQ board (National Instruments, NI USB-6343).

#### Stimulation

Stimuli were applied to glabrous skin in the following order: airpuff (Dust-Off, DPSXL4A), brush (small paintbrush), von Frey filaments (in order of increasing force; North Coast Medical, NC12775), poking with closed forceps (FST, 11050–10), controlled indentation with a mechanical stimulator, pinch, and temperature stimuli. Pinch and temperature stimuli were then applied to the thigh.

For controlled indentation, an indenter (Aurora Scientific, 300C-I) with a custom-made 200 μm diameter tip was used. The paw was first stimulated manually to identify spots that gave rise to robust calcium responses, and an indenter was then placed on top of that spot. Multiple spots were tested. A custom-written MATLAB program was used to design and trigger the stimuli. Baseline was recorded for 2 s, indentation lasted for 4 s, and 6 s of decay time was allowed. The interstimulus interval was 10 seconds. Each force was presented 3 times.

For thermal stimuli, a Peltier device (13*12*2.5mm, TE-65–0.6–0.8, TE technology) was controlled by a Temperature Controller (TEC1089/PT1000, Meerstetter Engineering) with an RTD Platinum (Pt) thermistor (2952-P1K0.161.6W.A.010-ND, Digikey) mounted on the surface of the Peltier. To maximize the conductivity between the Peltier and skin, a small amount of thermal paste (Aeronaut, Thermal Grizzly) was evenly applied on the top of the Peltier surface, which was then gently pressed on the skin until good contact was formed. A thermocouple microprobe (Physitemp, IT-1E) was inserted between the Peltier device and the skin as a separate measurement of the applied temperature. Thirty seconds after contacting skin, temperature stimuli started from innocuous temperatures progressing to noxious temperatures, in the sequence of 35, 25, 20, 40, 15, 45, 10, 50, 5, 55°C. Stimulus structure was as follows:

1. Baseline (32°C) - 20 seconds
2. Target temperature - 20 seconds
3. Baseline - 50 seconds
4. Back to 1.

The change rate for all temperatures was kept at 5°C s^−1^.

### Calcium imaging analysis

The analysis was performed as previously described^27^. Motion correction and spatial high-pass filtering were conducted using a custom-written macro code that employed the ImageJ plugin “moco” and “Unsharp mask” filter, then regions of interest (ROIs) were manually selected in ImageJ. Intensity measurements were then analyzed in MATLAB with a custom-written code. In the calculation of ΔF/F, F was defined using baseline activity (average intensity before each stimulation). When a cell responded to controlled indentation of multiple spots, responses with the lowest force threshold were selected. Responses to three trials of force presentation were averaged, and the average trace was used for threshold and intensity calculations. Threshold was determined to be the lowest force that evoked a discernible calcium response. Response intensity was calculated as ΔF/F area under the curve (AUC) for 3 seconds of indentation. For the calculation of the percentage of cells responding to a given stimulus for Figure 2C, the number of cells responding to pinch was used as a denominator.

### *In vivo* loose patch targeted DRG recordings

The recordings were performed as previously described^89^. Mice were treated with dexamethasone (2 mg/kg i.p.) and anesthetized with an i.p. injection of urethane (1 g/kg); anesthesia was maintained with 1–1.5% inhaled isoflurane. The L4 DRG was exposed similarly to the procedure described in the calcium imaging section. The exposed DRG was immersed in external solution containing 140 mM NaCl, 3.1 mM KCl, 0.5 mM KH_2_PO_4_, 6 mM glucose, 1.2 mM CaCl_2_, 1.2 mM MgSO_4_ (pH adjusted to 7.4 with NaOH); the same solution was used to fill glass pipettes with a 20–30 μm tip diameter. Fluorescent cell bodies that were labeled with a genetic reporter and CTB-A555 were targeted for loose-seal cell-attached recordings. Extracellular action potentials were measured using a Multiclamp 700A amplifier (Axon Instruments) operated in the voltage clamp configuration. Electrophysiological data were digitized at 40 kHz with a Digidata 1550a (Molecular Devices), low-pass filtered at 10 kHz (four-pole Bessel filter), and acquired using pClamp (Molecular Devices, Version 10). Stimuli were delivered manually or with a mechanical stimulator, as described in the calcium imaging section. A 250 μm diameter indenter tip was used for controlled indentation. For optogenetic stimulation, the same equipment was used as described in the optical activation behavior section. Electrical stimuli were applied using a bipolar electrode to glabrous skin moistened with 0.9% saline. Conduction velocity was measured by electrically stimulating the skin and dividing the distance between stimulation site and cell body by the spike latency. Electrophysiological data were analyzed using custom-made Python code as previously described^89^.

### *In vivo* DRG recordings with sharp glass electrodes

The recordings were performed as previously described^100^. Mice were anesthetized with urethane (1.5 g/kg). L4 DRG was exposed similarly to how described in the calcium imaging section. 1 MΩ glass electrode filled with 0.9% saline was advanced into the right L4 DRG while optical search stimulus was applied to the glabrous hindpaw. Controlled indentations were applied using a custom-built mechanical stimulator as previously described. Electrical stimuli were applied using a bipolar electrode to glabrous skin moistened with 0.9% saline. Conduction velocity was measured by electrically stimulating the skin and dividing the distance between stimulation site and cell body by the spike latency. To test the presence of DCN-projecting collaterals, the DCN were exposed similar to how described in the retrograde labeling section. An optical fibre (400 μm diameter, 0.39 NA) was placed above the DCN, and blue light pulses were delivered to generate antidromic spikes (Thorlabs, M470F3). Recordings were amplified using a Multiclamp 700B commander (Molecular Devices) under the 100X AC differential amplification mode with an additional 20X gain. Data were collected at 20 kHz using a Digidata 1550B (Molecular Devices).

### Spinal cord MEA recordings

The recordings and subsequent analysis were performed as previously described^53^. Briefly, mice were anesthetized with urethane (1.5 g/kg i.p.) and their body temperature was maintained at 37°C throughout the experiment. After exposure, the T13 vertebra was retracted to reveal the dorsal surface of the L4-L5 segments of spinal cord. The dura was removed, and the spinal cord was covered with 0.9% saline. A 32-channel silicon probe (Neuronexus Cambridge Neurotech ASSY-37 H4 optrode) was inserted into the spinal cord and advanced up to ~700 μm below the dorsal surface under visual guidance. Signals were amplified, filtered (0.1 – 7.5 kHz bandpass), and digitized (20 kHz) using a headstage amplifier and recording controller (Intan Technologies RHD2000 and Recording Controller). Data acquisition was controlled with open-source software (Intan Technologies Recording Controller version 2.07). Spike sorting was performed using JRCLUST.

### Whole cell recordings

#### Retrograde injections

Three days prior to the experiment, 200 nl of 5 mg/μL CTB-A555 was injected bilaterally into the PBN of *Smr2*^*Cre*^*; Calca-FlpE; R26*^*FSF-LSL-ReaChR::mCitrine*^ mice aged P14–18. Whole-cell patch-clamp recordings were then performed on CTB^+^ neurons while optically stimulating the ReaChR-containing afferents, as previously described^73^.

#### Slice preparation

Mice (P17–21) were anesthetized with inhaled isoflurane (4% – 5%) while vertebral columns were dissected. Lumbar enlargements were dissected out from vertebral columns in an ice-cold choline solution (92 mM Choline Chloride, 2.5 mM KCl, 1.2 mM NaH_2_PO_4_, 30 mM NaHCO_3_, 20 mM HEPES, 25 mM Glucose, 5 mM Sodium Ascorbate, 2 mM Thiourea, 3 mM Sodium Pyruvate, 10 mM MgSO_4_*7H_2_O, 0.5 mM CaCl_2_*2H_2_O, pH = 7.3–7.4, Osm = 300–310 mOsm) including 1 μM (*R*,*S*)-3-(2-carboxypiperazin-4-yl)propyl-1-phosphonic acid (CPP; Abcam, ab120160) and 1 μM 2,3-dihydroxy-6-nitro-7-sulfamoyl-benzo[f]quinoxaline (NBQX; Abcam, ab120046) and embeded in 5% LMP agarose (Life Technology, 16520–100). The lumbar spinal cords were sliced in a transverse plane (300 μm) (Leica, VT1200S), and the slices were recovered at 34°C for 30 minutes in oxygenated (95% O2 and 5% CO2) holding solution (119 mM NaCl, 2.5 mM KCl, 1.0 mM NaH_2_PO_4_*H2O, 26 mM NaHCO_3_, 25 mM Glucose, 1.3 mM Sodium Ascorbate, 2 mM MgSO_4_*7H_2_O, 2 mM CaCl_2_*2H_2_O, pH = 7.3–7.4, Osm = 300–310 mOsm). After recovery, spinal cord slices were placed at room temperature for 30 minutes prior to recordings.

#### Whole-cell recording procedure

Spinal cord slices were placed in a recording chamber maintained at room temperature and perfused with oxygenated (95% O2 and 5% CO2) recording ACSF (2.5 mM CaCl_2_*2H_2_O, 1.0 mM NaH_2_PO_4_*H2O, 119 mM NaCl, 2.5 mM KCl, 1.3 mM MgSO_4_*7H_2_O, 26 mM NaHCO_3_, 25 mM Glucose, 1.3 mM Sodium L-ascorbate, pH = 7.3–7.4, Osm = 300–310 mOsm). Retrogradely labeled SPB neurons were visualized using a SliceScope Pro 6000 electrophysiology rig (Scientifica) equipped with IR-DIC optics (ThorLabs, M850L3-C4, with COP1-B Collimation Adapter), an LED to fluorescently identify CTB^+^ cells (pE-300, Nikon CoolLED), a water-immersion objective (Olympus LUMPlanFLN 40X), and a camera (Teledyne QImaging, Rolera Bolt^™^ CMOS camera). For the cells within deep laminae, fluorescence from ReaChR::mCitrine^+^ afferents was used as a guide to target neurons in nests, i.e. surrounded by fluorescent axons. The pipette resistance ranged from 3 to 5 MΩ, and the electrodes were filled with an intracellular solution containing biocytin (130 mM Cs-gluconate, 10 mM HEPES, 1 mM EGTA, 0.1 mM CaCl_2_, 5 mM TEA, 1 mM QX-314, 0.2 D600, 0.4% w/v biocytin, pH 7.3, 295 mOsm). To avoid biocytin spillover, pipettes were front-filled with intracellular solution not containing biocytin. Whole-cell capacitance was left uncompensated (~5 pF) and the series resistance was constantly monitored and compensated online (60–80%). Signals were acquired using a Multiclamp 700A amplifier (Molecular Devices). The data were low-pass filtered at 2 kHz and digitized at 20 kHz (Molecular Devices, Digidata 1440A). For ReaChR-assisted circuit mapping, primary afferent synaptic terminals were stimulated with wide-field white LED illumination with a TxRd filter through the 40x objective (pE-300, Nikon CoolLED, 5 ms pulse width, light intensity = 12.35 mW). Current amplitude, latency, and jitter were analyzed using Clampfit (version 10, Molecular Devices). Cells were recorded first without additional drugs, and then in a solution containing 1 μM tetrodotoxin (TTX) (Tocris, 1069), followed by the addition of 500 μM 4-aminopyridine (4-AP) (Tocris, 940) to the bath.

#### Cell fills

Recordings were maintained for 40 minutes to allow biocytin to perfuse the cell. Subsequently, the slices were fixed in 4% PFA at 4°C overnight and processed for immunohistochemistry. Briefly, slices were washed in 0.3% TritonX-100 in 1X PBS 5 times for 30 minutes, then transferred to blocking solution (5% normal donkey serum, 0.3% TritonX-100 in 1X PBS) for 2 hours at room temperature. Slices were then incubated in blocking solution with primary antibodies for 72 hours, washed as described above and incubated in blocking solution with secondary antibodies for 48 hours. Slices were washed again and then dehydrated with serial incubation in 50%, 75%, and 100% methanol in PBS at room temperature for 10 minutes each and kept in 100% methanol overnight for full dehydration. Before imaging, slices were cleared in BABB solution for 2 hours.

### Quantification and statistical analysis

Statistical analyses were performed in GraphPad Prism 10.2.3 and MATLAB. Data normality was tested with the Shapiro-Wilk test. Comparison between two independent groups was done with unpaired t-test (normally distributed data) and Mann-Whitney test (non-parametric data). Comparison between more than two independent groups was done with one-way ANOVA with Tukey’s multiple comparisons post-hoc test. A permutation test on the mean difference was used to compare calcium response intensity distributions. A p-value ≤ 0.05 was considered statistically significant.

## Supplementary Material

Supplement 1

Supplement 2

Supplement 3

Supplement 4

Supplement 5

Supplement 6

Supplement 7

## Figures and Tables

**Key resources table:** 

REAGENT or RESOURCE	SOURCE	IDENTIFIER
**Antibodies**		
Goat anti-mCherry (1:500, IHC)	OriGene	RRID:AB_2333093
Rabbit anti-dsRed (1:500, IHC)	Takara Bio	RRID:AB_10013483
Chicken anti-RFP (1:500, IHC)	Rockland	RRID:AB_10703148
Goat anti-GFP (1:500, IHC)	US Biological Life Sciences	G8965-01E
Chicken anti-NFH (1:500, IHC, skin)	Aves Labs	RRID:AB_2313552
Rabbit anti-NFH (1:500, IHC, DRG)	Sigma-Aldrich	RRID:AB_477272
Rabbit anti-CGRP (1:500, IHC)	Immunostar	RRID:AB_572217
Goat anti-TrkA (1:500, IHC)	R&D Systems	RRID:AB_2283049
Rabbit anti-CalB (1:5000, IHC)	Swant	RRID:AB_3107026
Rabbit anti-TrpV1 (1:500, IHC)	Alomone Labs	RRID:AB_2313819
Rabbit anti-P2X3 (1:500, IHC)	Alomone Labs	RRID:AB_2313760
Rabbit anti-S100 (1:500, IHC)	VWR/ProteinTech	RRID:AB_2254244
Rabbit anti-NeuN (1:500, IHC)	Millipore	RRID:AB_10807945
Goat anti-CD31	R&D Systems	RRID:AB_2161028
IB4 (Alexa Fluor 488 conjugated)	Thermo Fisher	I21411
IB4 (Alexa Fluor 647 conjugated)	Thermo Fisher	I32450
Donkey anti-goat (Alexa Fluor 546)	Thermo Fisher	RRID:AB_2534103
Donkey anti-goat (Alexa Fluor 647)	Thermo Fisher	RRID:AB_11192020
Donkey anti-goat (Alexa Fluor 488)	Thermo Fisher	RRID:AB_2534102
Donkey anti-rabbit (Alexa Fluor 546)	Thermo Fisher	RRID:AB_2534016
Donkey anti-rabbit (Alexa Fluor 647)	Thermo Fisher	RRID:AB_2536183
Donkey anti-rabbit (Alexa Fluor 488)	Thermo Fisher	RRID:AB_2535792
Donkey anti-chicken (Alexa Fluor 555)	Thermo Fisher	RRID:AB_2921071
Donkey anti-chicken (Alexa Fluor 488)	Thermo Fisher	RRID:AB_2921070
Donkey anti-chicken (Alexa Fluor 647)	Jackson	RRID:AB_2340380
**Bacterial and virus strains**		
AAV9-CAG-FLEX-hPLAP	BCH viral core	N/A
AAV2/9-CAG-CreON-FlpON-SEAP	BCH viral core	N/A
AAV2-retro-hSyn-FlpO	BCH viral core	N/A
rAAV1/2-Cre virus	Penn Viral Core	N/A
**Chemicals, peptides, and recombinant proteins**		
Urethane	Sigma	U2500
Isoflurane	Covetrus	029405
Tamoxifen	Sigma	T5648-1g
Paraformaldehyde (PFA), reagent grade, crystalline	Sigma	P6148-500G
Picric acid-formaldehyde (PAF) fixative (Zamboni)	Fisher	NC9335034
Normal Donkey Serum	Jackson	017-000-121
Benzyl alcohol	Sigma	108006-500 ml
Benzyl benzoate 99%	Fisher	AC105860010
OCT	Fisher	1437365
Fluoromount-G	Southern Biotech	0100-01
DAPI-Fluoromount-G	Southern Biotech	0100-20
CTB-Alexa Fluor(R) 555 conjugate	Fisher	C34776
CTB-Alexa Fluor(R) 647 conjugate	Fisher	C34778
BCIP	Sigma	11383221001
NBT	Sigma	11383213001
**Critical commercial assays**		
RNAscope^™^ Multiplex Fluorescent Reagent Kit v2	ACD bio	323100
**Experimental models: Organisms/strains**		
Mouse: *Smr2*^*Cre*^	Jax, Qi et al.^27^	RRID:IMSR_JAX:039562
Mouse: *Bmpr1b*^*Cre*^	Jax, Sharma et al.^35^	RRID:IMSR_JAX:039561
Mouse: *Sstr2*^*CreER*^	Jax, Qi et al.^27^	RRID:IMSR_JAX:039563
Mouse: *MrgprD*^*CreER*^	Jax, Olson et al.^37^	RRID:IMSR_JAX:031286
Mouse: *Cysltr2*^*Cre*^	Jax, Qi et al.^27^	RRID:IMSR_JAX:039985
Mouse: *TrpmpF*^*FlpO*^	Jax, Qi et al.^27^	RRID:IMSR_JAX:039564
Mouse: *TrkB*^*CreER*^	Jax, Rutlin et al.^38^	RRID:IMSR_JAX:027214
Mouse: *R26*^*LSL-FSF-tdTomato*^	Jax, Madisen et al.^90^	RRID:IMSR_JAX:021875
Mouse: *R26*^*LSL-FSF-ReaChR::mCitrine*^	Jax, Madisen et al.^91^	RRID:IMSR_JAX:024846
Mouse: *R26*^*LSL-ReaChR::mCitrine*^	Jax	RRID:IMSR_JAX:026294
Mouse: *R26*^*LSL-tdTomato*^	Jax, Madisen et al.^91^	RRID:IMSR_JAX:007914
Mouse: *TIGRE*^*LSL-GCaMP6f*^	Jax, Daigle et al.^92^	RRID:IMSR_JAX:030328
Mouse: *TIGRE*^*LSL-FSF-GCaMP7s*^	Jax	RRID:IMSR_JAX:034112
Mouse: *Tau*^*FSFiAP*^	Jax, Lehnert et al.^93^	RRID:IMSR_JAX:039981
Mouse: *MrgprB4*^*Cre*^	Jax, Vrontou et al.^39^	RRID:IMSR_JAX:021077
Mouse: *MrgprA3*^*Cre*^	Han et al.^40^	N/A
Mouse: *Calca-FlpE*	Choi et al.^73^	N/A
Mouse: *Avil*^*FlpO*^	Choi et al.^73^	N/A
Mouse: *Calca*^*iDTR*^	McCoy et al.^94^	N/A
Mouse: *Ptgfr*^*LreFR*^	This study	N/A
**Oligonucleotides**		
mm-Bmpr1b-C3	ACD bio	533941-C3
mm-Smr2-C2	ACD bio	538931-C2
mm-Calca	ACD bio	420361
mm-Piezo2-C3	ACD bio	1228811-C3
GCaMP	ACD bio	557091
**Software and algorithms**		
MATLAB	Mathworks	RRID: SCR_001622
Python	The Python Software Foundation	RRID:SCR_008394
FIJI	NIH	RRID:SCR_002285
Arduino IDE	Arduino	RRID:SCR_024884
GraphPad Prism	GraphPad	RRID:SCR_002798
ZEISS ZEN Microscopy Software	Zeiss	RRID: SCR_013672
