# Supplementary material for: Fast-conducting mechanonociceptors uniquely engage reflexive and affective pain circuitry to drive protective responses
Source: bioRxiv. 2025 Nov 20:2025.11.11.687663. Preprint. [Version 2] doi: 10.1101/2025.11.11.687663 (PMC12642651; doi:10.1101/2025.11.11.687663)
Supplement: Supplement 7 [file NIHPP2025.11.11.687663v2-supplement-7.pdf]

bioRxiv preprint doi: https://doi.org/10.1101/2025.11.11.687663; this version posted November 20, 2025. The copyright holder for this preprint (which was not certified by peer review) is the author/funder, who has granted bioRxiv a license to display the preprint in perpetuity. It is made available under aCC-BY 4.0 International license.

Supplemental Figure Legends

bioRxiv preprint doi: https://doi.org/10.1101/2025.11.11.687663; this version posted November 20, 2025. The copyright holder for this preprint (which was not certified by peer review) is the author/funder, who has granted bioRxiv a license to display the preprint in perpetuity. It is made available under aCC-BY 4.0 International license.

Figure S1. Optically evoked activity in the spinal cord in the absence of an observable behavioral response, related to Figure 1

(A) *In vivo* multielectrode array recordings from lumbar spinal cord neurons. Sensory neurons are stimulated with light pulses on the glabrous skin of animals expressing ReaChR in different DRG populations.

(B) Percentage of recorded units with light-evoked responses. Units are summed across multiple animals (n = 2, 1, 3 animals from left to right).

(C) Light stimulation of the paw does not evoke reliable withdrawal. Average (mean ± SD) withdrawal frequency plotted. Longer stimulation (500 ms 0.2 Hz pulses for *MrgprA3*^{Cre}*; R26*$^{LSL\text{-}ReaChR}$, *Cysltr2*^{Cre}*; R26*$^{LSL\text{-}ReaChR}$ and littermate controls that lack a driver or ReaChR allele, continuous light for *MrgprB4*^{Cre}*; R26*$^{LSL\text{-}ReaChR}$) does not increase withdrawal frequency (see Figure 1C for 5-ms pulse stimulation). Each dot represents an animal (n = 5, 8, 4, 5 from left to right).

(D) Example spike rasters and peristimulus time histograms (PSTHs) from three representative units from *MrgprA3*^{Cre}*; R26*$^{LSL\text{-}ReaChR}$ and *Cysltr2*^{Cre}*; R26*$^{LSL\text{-}ReaChR}$ animals, 2 units from an *MrgprB4*^{Cre}*; R26*$^{LSL\text{-}ReaChR}$ animal. Stimulus is 500 ms light pulse.

Figure S2. Characterization of Aβ-HTMRs labeled by the *Ptgfr*^{CreER} mouse line, related to Figure 1

(A) Immunostaining of lumbar DRG sections from mice with genetically labeled *Ptgfr*$^{+}$ neurons. Note the large diameter of reporter^{+} cells. Scale bars are 50 µm.

(B) Average percentage (mean ± SD) of reporter^{+} neurons that co-express a given marker. Each dot represents an average of several DRG sections from a single animal (n = 3, 2 mice from left to right).

(C) Comparative characterization of Aβ-LTMRs (labeled broadly with *Avil*^{FlpO}, units selected based on fast conduction velocity and low mechanical threshold), Aβ-HTMRs (labeled with *Ptgfr*^{CreER}), and Aδ-HTMRs (labeled broadly with *Calca-FlpE*, only signals from A-fiber neurons are picked up with this technique) using *in vivo* electrophysiological recordings with

bioRxiv preprint doi: https://doi.org/10.1101/2025.11.11.687663; this version posted November 20, 2025. The copyright holder for this preprint (which was not certified by peer review) is the author/funder, who has granted bioRxiv a license to display the preprint in perpetuity. It is made available under aCC-BY 4.0 International license.

sharp glass electrodes in anesthetized mice. Representative voltage responses to optical stimulation of the skin or the dorsal column nuclei (DCN), and electrical stimulation of the skin.

(D) Representative responses of the three sensory neuron populations to mechanical stimulation of the glabrous paw skin with precise indentation using a 200 μm probe tip.

(E) Conduction velocity measurements for the three sensory neuron populations. Each dot represents a single unit (n = 9 units from 6 animals for Aβ-LTMRs, 11 units from 5 animals for Aβ-HTMRs, 11 units from 4 animals for Aδ-HTMRs).

(F) Indentation force threshold measurement for the three sensory neuron populations. Each dot represents a single unit (n = 15 units from 10 animals for Aβ-LTMRs, 8 units from 4 animals for Aβ-HTMRs, 11 units from 4 animals for Aδ-HTMRs).

(G) Percentage of recorded units that have axons projecting to the DCN, determined by antidromic stimulation (7/8 units for Aβ-LTMRs, 12/13 units for Aβ-HTMRs, 0/9 units for Aδ-HTMRs).

(H) Sections of spinal cord dorsal horn from a $Ptgfr^{CreER}$; $Avil^{FlpO}$; $R26^{LSL\text{-}FSF\text{-}tdTomato}$ animal. The genetically labeled Aβ-HTMRs terminate predominantly in the deep dorsal horn, below the IB4 band. The terminals are dense and diffuse, distinct from the clustered pattern of the $CGRP^{+}$ axons. Scale bars are 100 μm.

(I) Sections of glabrous paw skin from a $Ptgfr^{CreER}$; $Avil^{FlpO}$; $R26^{LSL\text{-}FSF\text{-}tdTomato}$ animal. The genetically labeled Aβ-HTMRs are $CGRP^{-}$ and terminate in the epidermis. Scale bars are 50 μm.

(J) Examples of whole-mount AP staining of peripheral arbors of DCN-projecting Aβ-HTMRs. AAV-Flp virus was injected into the DCN of $Ptgfr^{CreER}$; Tau^{FSFiAP} animals to achieve sparse labeling. Individual arbors are large compared to those in (K). Scale bars are 200 μm.

(K) Examples of whole-mount AP staining of peripheral arbors of DCN-projecting Aβ-LTMRs. AAV-Cre virus was injected into the DCN of $Brn3a^{AP}$ animals to achieve sparse labeling. LTMR identity of labeled neurons is inferred based on known morphology. Scale bars are 200 μm.

(L) Sections of glabrous palm skin from a human donor. Arrows point to $PGP9.5^{+}CGRP^{-}$ endings in the epidermis that are NFH^{+} in the dermis, reflecting myelination. Scale bars are 50 μm.

bioRxiv preprint doi: https://doi.org/10.1101/2025.11.11.687663; this version posted November 20, 2025. The copyright holder for this preprint (which was not certified by peer review) is the author/funder, who has granted bioRxiv a license to display the preprint in perpetuity. It is made available under aCC-BY 4.0 International license.

Figure S3. Additional physiological characterization of the genetically labeled Aδ-HTMRs and C-heat thermoreceptors, related to Figure 2

(A) Calcium indicator responses of *Smr2*^{Cre}- and the *Bmpr1b*^{Cre}-labeled neurons to indentation of glabrous skin with von Frey filaments. While both populations only respond to relatively high forces, above 1 g, *Bmpr1b*^{Cre}-labeled neurons are more sensitive than *Smr2*^{Cre}-labeled neurons (n = 223 *Smr2*^{Cre}-labeled neurons across 9 animals, 246 *Bmpr1b*^{Cre}-labeled neurons across 3 animals).

(B) Calcium responses of *Smr2*^{Cre}-labeled neurons to controlled indentation of glabrous skin in Piezo2 conditional knockout (cKO) mice and littermate controls. *Smr2*^{Cre}*; Piezo2*$^{null/fl}$*; TIGRE*$^{LSL\text{-}GCaMP6f}$ mice were used as Piezo2 cKO, littermates with one floxed and one wild-type *Piezo2* allele were used as controls (n = 39 neurons across 5 animals for control, and 49 neurons across 6 animals for Piezo2 cKO).

(C) Indentation force threshold (mean ± SD) of *Smr2*^{Cre}-labeled neurons is unaffected by Piezo2 knockout. Each dot/triangle represents a cell (n same as in (B), Mann-Whitney test).

(D) Indentation response intensity (mean ± SD) of *Smr2*^{Cre}-labeled neurons is unaffected by Piezo2 knockout. A quantification of calcium responses shown in (B). Each dot/triangle represents an average intensity value from n cells (n same as in (B), permutation test on the mean difference, 10000 permutations).

(E) *Piezo2* expression is very low in *GCaMP*$^{+}$ *Smr2*^{Cre}-labeled neurons compared to other DRG neurons in both Piezo2 cKO and control mice. Representative images of RNAScope on DRG sections. Scale bars are 50 µm.

(F-G) Temperature responses of *Smr2*^{Cre}- and the *Bmpr1b*^{Cre}-labeled neurons vary by innervation target. Calcium responses of *Smr2*^{Cre}- and the *Bmpr1b*^{Cre}-labeled neurons to different temperatures presented to (F) glabrous paw and (G) hairy thigh skin. Pinch responses are also shown for comparison. The hairy skin-innervating *Smr2*^{Cre}-labeled neurons are more heat-sensitive than their glabrous skin-innervating counterparts. On the other hand, the glabrous skin-innervating *Bmpr1b*^{Cre}-labeled neurons are more heat-sensitive than their hairy skin-innervating counterparts (n = 110 glabrous skin-innervating and 126 hairy skin-innervating *Smr2*^{Cre}-labeled

bioRxiv preprint doi: https://doi.org/10.1101/2025.11.11.687663; this version posted November 20, 2025. The copyright holder for this preprint (which was not certified by peer review) is the author/funder, who has granted bioRxiv a license to display the preprint in perpetuity. It is made available under aCC-BY 4.0 International license.

neurons from 15 animals, 140 glabrous skin-innervating and 12 hairy skin-innervating *Bmpr1b*^{Cre}-labeled neurons from 3 animals).

(H) *Sstr2*^{CreER}-labeled neurons are tuned to noxious heat. Calcium responses of *Sstr2*^{CreER}-labeled neurons to select temperatures and pinch (n = 37 *Sstr2*^{CreER}-labeled neurons from 3 animals).

(I) Aδ-HTMRs have wide extracellular spike waveforms compared to Aβ-LTMRs. Example spike waveforms acquired with *in vivo* loose patch electrophysiology. Each waveform is from a different unit. The Aβ-LTMR example is replotted from Emanuel et al. 2021[89].

Figure S4. Molecular characterization of genetically labeled Aδ-HTMRs, related to Figure 2

(A-B) Immunostaining of lumbar DRG sections from mice with genetically labeled (A) *Smr2*$^{+}$ and (B) *Bmpr1b*$^{+}$ neurons. Scale bars are 50 µm.

(C) *Smr2*^{Cre}- and *Bmpr1b*^{Cre}-labeled neurons have medium/large-diameter soma, larger than many other CGRP^{+} neurons. Diameters of reporter^{+} or marker^{+} soma are plotted (n = 237, 183, 369, 176, 580 cells across 2, 2, 1, 3, 3 animals from left to right).

(D) Together, the *Smr2*^{Cre}- and *Bmpr1b*^{Cre}- labeled populations account for most large-diameter CGRP^{+} cells. Average percentage (mean ± SD) of all and large diameter CGRP^{+} cells that are *Smr2*^{Cre}- or *Bmpr1b*^{Cre}-labeled. Large diameter is defined as > 25 µm. Each dot represents an animal (n = 3 animals for all analyses).

(E) Average percentage (mean ± SD) of reporter^{+} neurons that co-express a given marker. Each dot represents an average of 10 DRG sections from a single animal (n = 3 animals for all analyses).

Figure S5. Chemogenetic ablation of Aδ-HTMRs, related to Figure 3

(A) Examples of facial wounds developed by mice whose Aδ-HTMRs were ablated.

(B) Chemogenetic ablation of Aδ-HTMRs is ~50% efficient. Average number (mean ± SD) of *Smr2*$^{+}$ and *Bmpr1b*$^{+}$ cells across experimental groups. Each dot represents an average of 10

bioRxiv preprint doi: https://doi.org/10.1101/2025.11.11.687663; this version posted November 20, 2025. The copyright holder for this preprint (which was not certified by peer review) is the author/funder, who has granted bioRxiv a license to display the preprint in perpetuity. It is made available under aCC-BY 4.0 International license.

DRG sections from a single animal (n = 9, 3, 6, 6 animals from left to right, ***$p \leq 0.001$, ****$p \leq 0.0001$, one-way ANOVA with Tukey's multiple comparisons post-hoc test).

Figure S6. Peripheral morphology of genetically labeled Aδ-HTMRs, related to Figures 4 and 5

(A) Axons from multiple Aδ-HTMRs overlap to create a dense network of endings in glabrous skin. Representative image pairs exemplifying the process of overlap index quantification. In each pair of images, the left image is of sparsely labeled Aδ-HTMR axons, achieved with low-titer reporter virus injection (see Methods for details), and the right image is of densely labeled Aδ-HTMRs, achieved with constitutive genetic labeling. The green outlines are the axon reconstructions. Scale bars are 500 μm.

(B) Genetically labeled Aδ-HTMRs penetrate the epidermis and form free nerve endings. Representative confocal images of *Smr2^{Cre}; Calca-FlpE; R26$^{LSL\text{-}FSF\text{-}tdT}$* and *Bmpr1b^{Cre}; Calca-FlpE; R26$^{LSL\text{-}FSF\text{-}tdT}$* glabrous skin sections. Reporter^{+} endings are pointed out using white arrows. Dashed lines indicate the border between the dermis and epidermis. Scale bars are 50 μm.

(C) Genetically labeled Aδ-HTMRs innervate the walls of the urinary bladder. Representative confocal images from *Smr2^{Cre}; Calca-FlpE; R26$^{LSL\text{-}FSF\text{-}tdT}$* and *Bmpr1b^{Cre}; Calca-FlpE; R26$^{LSL\text{-}FSF\text{-}tdT}$* whole-mount stained bladders. The reporter signal represents a part of the CGRP^{+} signal and can be observed throughout the tissue. Scale bars are 200 μm.

(D) Genetically labeled Aδ-HTMRs are found in the trigeminal ganglia. Representative confocal images of *Smr2^{Cre}; Calca-FlpE; R26$^{LSL\text{-}FSF\text{-}tdT}$* and *Bmpr1b^{Cre}; Calca-FlpE; R26$^{LSL\text{-}FSF\text{-}tdT}$* trigeminal ganglion sections. As in the DRG, reporter^{+} cells co-express CGRP and do not bind IB4. Scale bars are 200 μm.

Figure S7. Central termination patterns of genetically labeled Aδ-HTMRs, related to Figure 6

(A) A representative confocal image of the *Smr2^{Cre}; Bmpr1b^{Cre}; Calca-FlpE; R26$^{LSL\text{-}FSF\text{-}tdT}$* deep dorsal horn from a lumbar spinal cord section. This section was stained using both anti-tdTomato

bioRxiv preprint doi: https://doi.org/10.1101/2025.11.11.687663; this version posted November 20, 2025. The copyright holder for this preprint (which was not certified by peer review) is the author/funder, who has granted bioRxiv a license to display the preprint in perpetuity. It is made available under aCC-BY 4.0 International license.

and anti-CGRP antibodies and shows that most $CGRP^+$ axons are $tdTomato^+$. Scale bar is 50 µm.

(B) Representative confocal images of the control (wild type littermate) and Aδ-HTMR-ablated (*Smr2^{Cre}; Bmpr1b^{Cre}; Calca^{iDTR}*) dorsal horn from lumbar spinal cord sections. Note that the CGRP signal is drastically diminished in the animal subjected to Aδ-HTMR ablation. Scale bar is 100 µm.

(C) The majority of the CGRP signal overlaps with the Aδ-HTMR-reporter signal. Quantification related to the example shown in (A). Each dot represents a spinal cord section (n = 30 sections across 3 animals).

(D) The CGRP signal is significantly diminished in animals subjected to Aδ-HTMR ablation. Quantification related to the example shown in (B). The Aδ-HTMR-ablated group consists of *Smr2^{Cre}; Bmpr1b^{Cre}; Calca^{iDTR}* animals. Each dot represents a spinal cord section (n = 15 across 3 animals for controls, 10 across 2 animals for Aδ-HTMR-ablated group; $****p \leq 0.0001$, unpaired t test).

(E) Aδ-HTMRs endings form nests around large cell bodies in the deep dorsal horn of the spinal cord. Representative confocal images from *Smr2^{Cre}; Calca-FlpE; R26$^{LSL\text{-}FSF\text{-}tdT}$* spinal cord sections. Nests of Aδ-HTMR endings are pointed out with white arrows. Scale bars are 50 µm.

(F) Aδ-HTMR terminals are in close proximity to the lamina I anterolateral tract (ALT) projection neurons. Representative confocal images from *Smr2^{Cre}; Calca-FlpE; R26$^{LSL\text{-}FSF\text{-}tdT}$* and *Bmpr1b^{Cre}; Calca-FlpE; R26$^{LSL\text{-}FSF\text{-}tdT}$* spinal cord sections. ALT projection neurons were retrogradely labeled with CTB-A647 injection into the PBN. Scale bars are 20 µm.

Supplemental Video 1. Aδ-HTMRs evoke fast and robust nocifensive behavior. Behavioral response to optical activation of glabrous skin-innervating Aδ-HTMR in an *Smr2^{Cre}; Calca-FlpE; R26$^{LSL\text{-}FSF\text{-}ReaChR::mCitrine}$* mouse. Stimulus is a 5-ms light pulse. Video is acquired at 200 fps and replayed at 5 fps.

Supplemental Video 2. Pinprick paradigm. Pin is pushed up into the mouse paw with a button push. Video is acquired at 200 fps and replayed at 5 fps.

bioRxiv preprint doi: https://doi.org/10.1101/2025.11.11.687663; this version posted November 20, 2025. The copyright holder for this preprint (which was not certified by peer review) is the author/funder, who has granted bioRxiv a license to display the preprint in perpetuity. It is made available under aCC-BY 4.0 International license.

Supplemental Video 3. Aδ-HTMRs drive fast reflexive paw movement in spinalized animals. Behavioral response to optical activation of glabrous skin-innervating Aδ-HTMR in an *Smr2*^{Cre}*; Calca-FlpE; R26*$^{LSL\text{-}FSF\text{-}ReaChR::mCitrine}$ mouse after spinal transection. Mouse hindlimbs are paralyzed by the transection and pulled through the wire rack for easy optical access and unrestrained movement. Stimulus is a 5-ms light pulse. Video is acquired at 200 fps and replayed at 5 fps.

Supplemental Video 4. C-heat thermoreceptors drive slow reflexive paw movement in spinalized animals. Behavioral response to optical activation of glabrous skin-innervating C-heat thermoreceptors in an *Sstr2*^{CreER}*; R26*$^{LSL\text{-}ReaChR::mCitrine}$ mouse after spinal transection. Mouse hindlimbs are paralyzed by the transection and pulled through the wire rack for easy optical access and unrestrained movement. Stimulus is a 5-ms light pulse. Video is acquired at 200 fps and replayed at 5 fps.

Supplemental Video 5. C-heat thermoreceptors drive a unique delayed paw movement in spinalized animals. An example of extended behavioral response to optical activation of glabrous skin-innervating C-heat thermoreceptors in an *Sstr2*^{CreER}*; R26*$^{LSL\text{-}ReaChR::mCitrine}$ mouse after spinal transection. Note that the initial withdrawal-like movement is followed by delayed twisting of the paw in absence of additional stimulation. Stimulus is a 5-ms light pulse. Video is acquired at 200 fps and replayed at 25 fps.

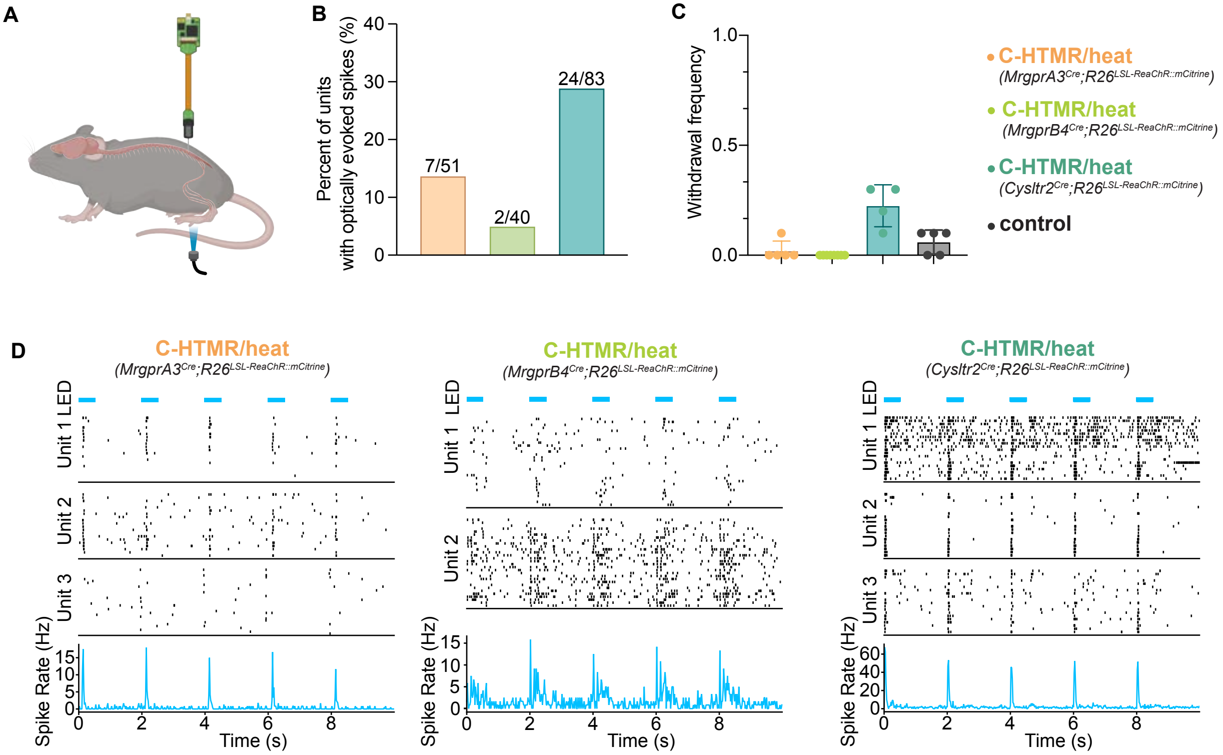
A
B
Percent of units with optically evoked spikes (%)
7/51
2/40
24/83
C
Withdrawal frequency
C-HTMR/heat (MrgprA3Cre;R26LSL-ReaChR::mCitrine)
C-HTMR/heat (MrgprB4Cre;R26LSL-ReaChR::mCitrine)
C-HTMR/heat (Cysltr2Cre;R26LSL-ReaChR::mCitrine)
control
D
LED
Unit 1
Unit 2
Unit 3
Spike Rate (Hz)
Time (s)

Figure S1

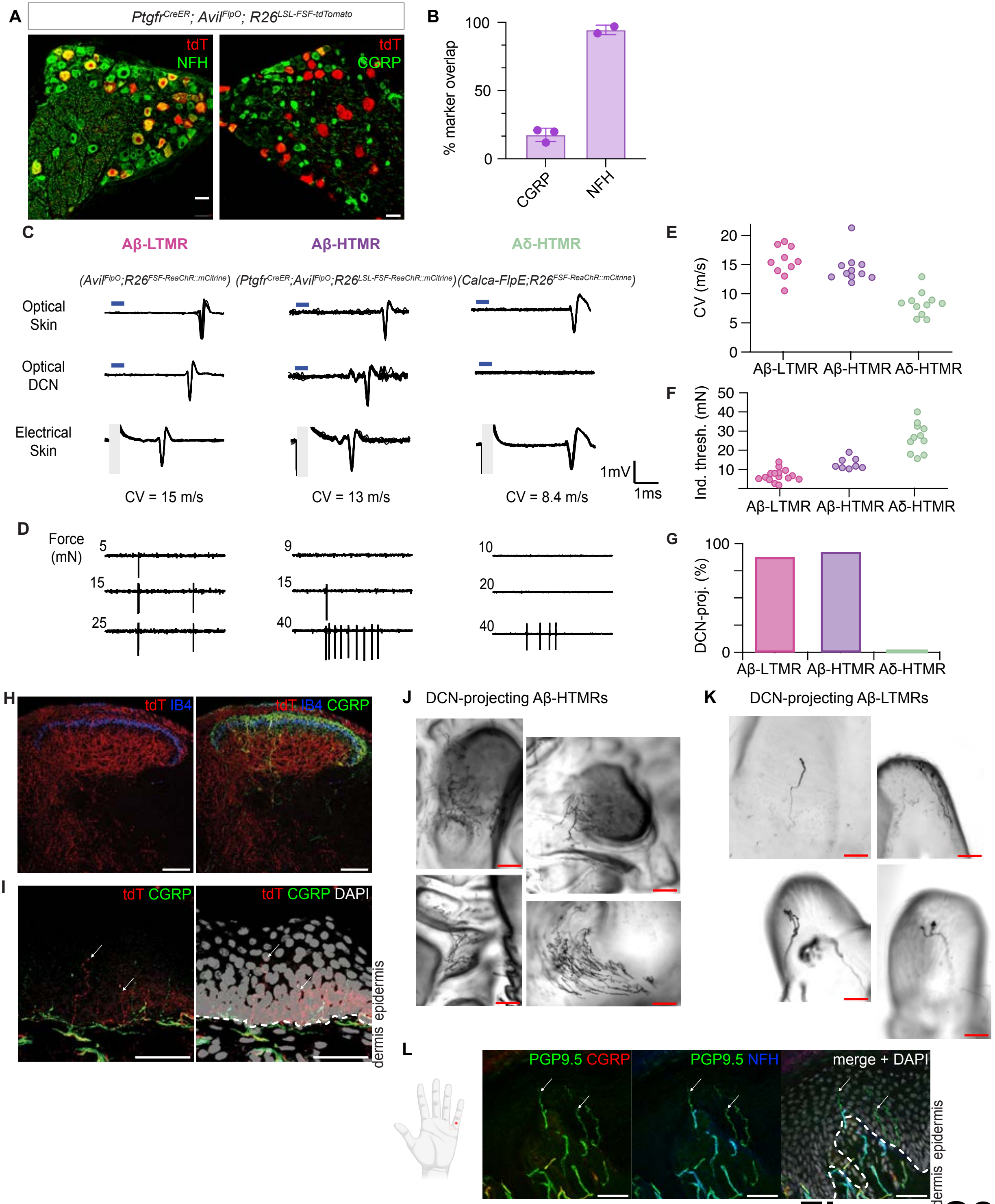

A
PtgfrCreER; AvilFlpO; R26LSL-FSF-tdTomato
tdT NFH
tdT CGRP
B
% marker overlap
100
50
0
CGRP
NFH
C
Aβ-LTMR
Aβ-HTMR
Aδ-HTMR
(AvilFlpO;R26FSF-ReaChR::mCitrine)
(PtgfrCreER;AvilFlpO;R26LSL-FSF-ReaChR::mCitrine)
(Calca-FlpE;R26FSF-ReaChR::mCitrine)
Optical Skin
Optical DCN
Electrical Skin
1mV
1ms
CV = 15 m/s
CV = 13 m/s
CV = 8.4 m/s
D
Force (mN)
5
15
25
9
15
40
10
20
40
E
CV (m/s)
20
15
10
5
0
Aβ-LTMR Aβ-HTMR Aδ-HTMR
F
Ind. thresh. (mN)
50
40
30
20
10
Aβ-LTMR Aβ-HTMR Aδ-HTMR
G
DCN-proj. (%)
100
0
Aβ-LTMR Aβ-HTMR Aδ-HTMR
H
tdT IB4
tdT IB4 CGRP
I
tdT CGRP
tdT CGRP DAPI
dermis epidermis
J
DCN-projecting Aβ-HTMRs
K
DCN-projecting Aβ-LTMRs
L
PGP9.5 CGRP
PGP9.5 NFH
merge + DAPI
dermis epidermis

Figure S2

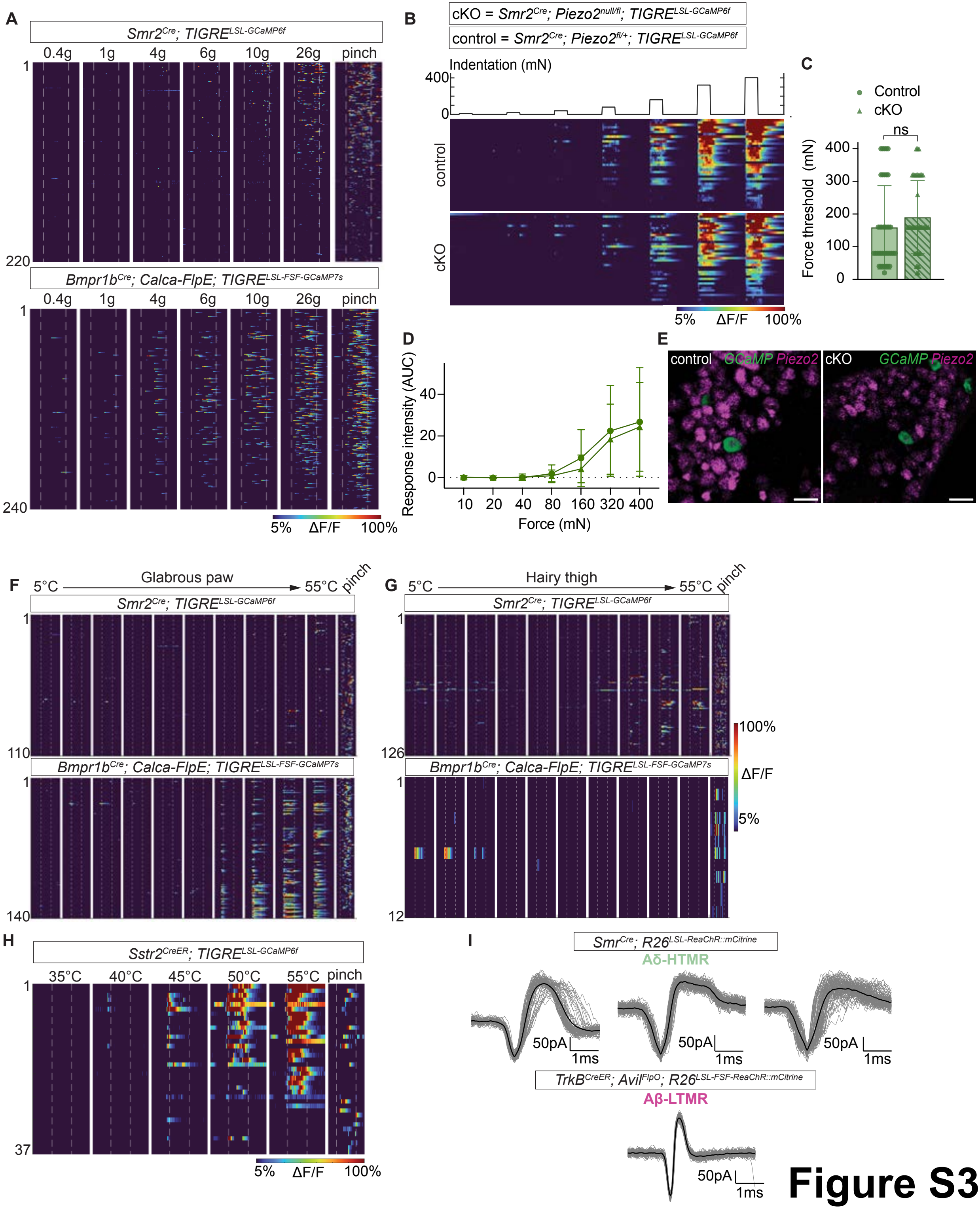
A
Smr2Cre; TIGRELSL-GCaMP6f
0.4g 1g 4g 6g 10g 26g pinch
1
220
Bmpr1bCre; Calca-FlpE; TIGRELSL-FSF-GCaMP7s
0.4g 1g 4g 6g 10g 26g pinch
1
240
5% ΔF/F 100%
B
cKO = Smr2Cre; Piezo2null/fl; TIGRELSL-GCaMP6f
control = Smr2Cre; Piezo2fl/+; TIGRELSL-GCaMP6f
Indentation (mN)
400
0
control
cKO
5% ΔF/F 100%
C
Control
cKO
ns
Force threshold (mN)
400
300
200
100
0
D
Response intensity (AUC)
40
20
0
10 20 40 80 160 320 400
Force (mN)
E
control GCaMP Piezo2
cKO GCaMP Piezo2
F
5°C Glabrous paw 55°C pinch
Smr2Cre; TIGRELSL-GCaMP6f
1
110
Bmpr1bCre; Calca-FlpE; TIGRELSL-FSF-GCaMP7s
1
140
G
5°C Hairy thigh 55°C pinch
Smr2Cre; TIGRELSL-GCaMP6f
1
126
Bmpr1bCre; Calca-FlpE; TIGRELSL-FSF-GCaMP7s
1
12
100%
ΔF/F
5%
H
Sstr2CreER; TIGRELSL-GCaMP6f
35°C 40°C 45°C 50°C 55°C pinch
1
37
5% ΔF/F 100%
I
SmrCre; R26LSL-ReaChR::mCitrine
Aδ-HTMR
50pA 1ms
50pA 1ms
50pA 1ms
TrkBCreER; AvilFlpO; R26LSL-FSF-ReaChR::mCitrine
Aβ-LTMR
50pA 1ms

Figure S3

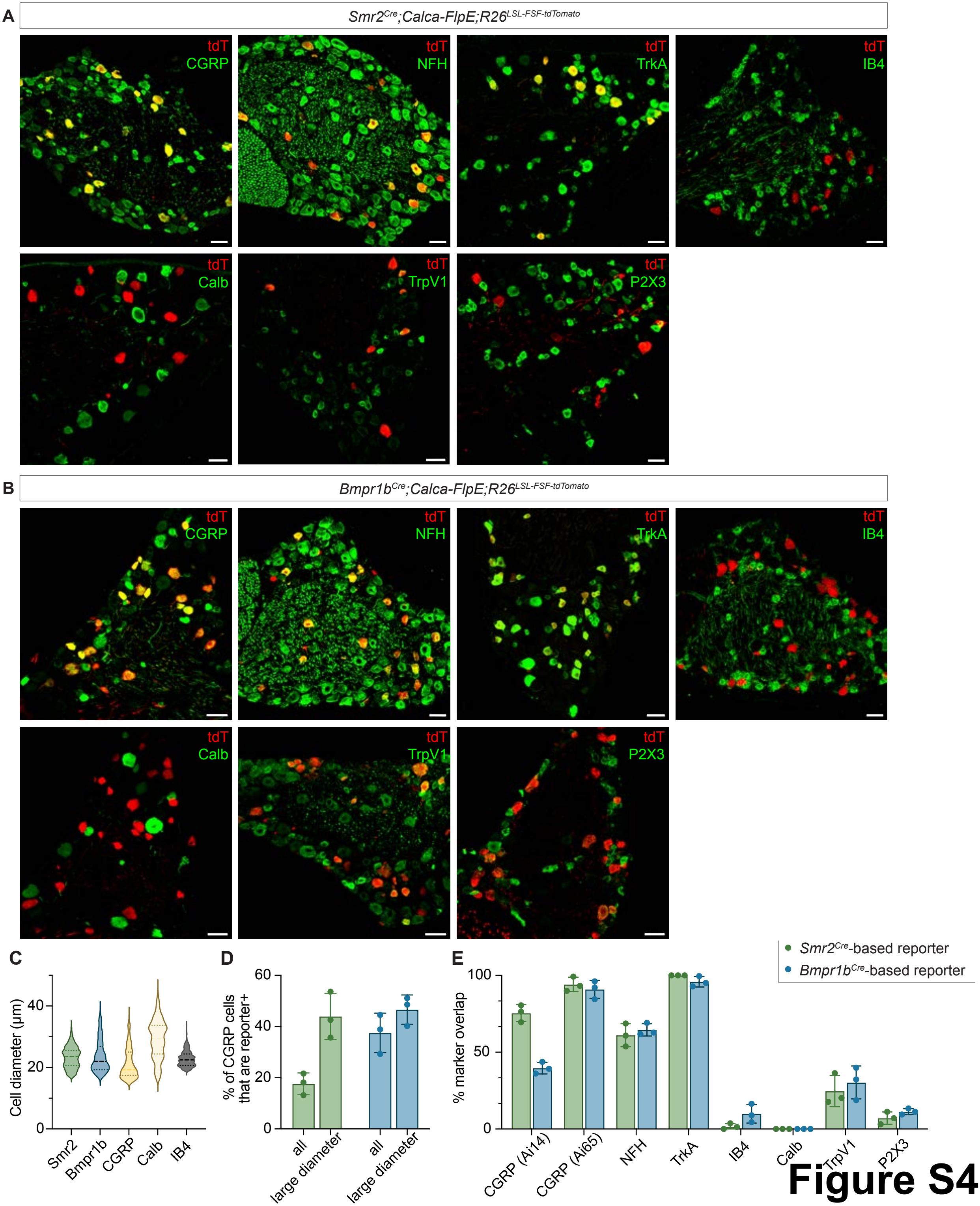
A
Smr2Cre;Calca-FlpE;R26LSL-FSF-tdTomato
tdT
CGRP
NFH
TrkA
IB4
Calb
TrpV1
P2X3
B
Bmpr1bCre;Calca-FlpE;R26LSL-FSF-tdTomato
C
Cell diameter (µm)
Smr2
Bmpr1b
CGRP
Calb
IB4
D
% of CGRP cells that are reporter+
all
large diameter
E
% marker overlap
CGRP (Ai14)
CGRP (Ai65)
Smr2Cre-based reporter
Bmpr1bCre-based reporter

Figure S4

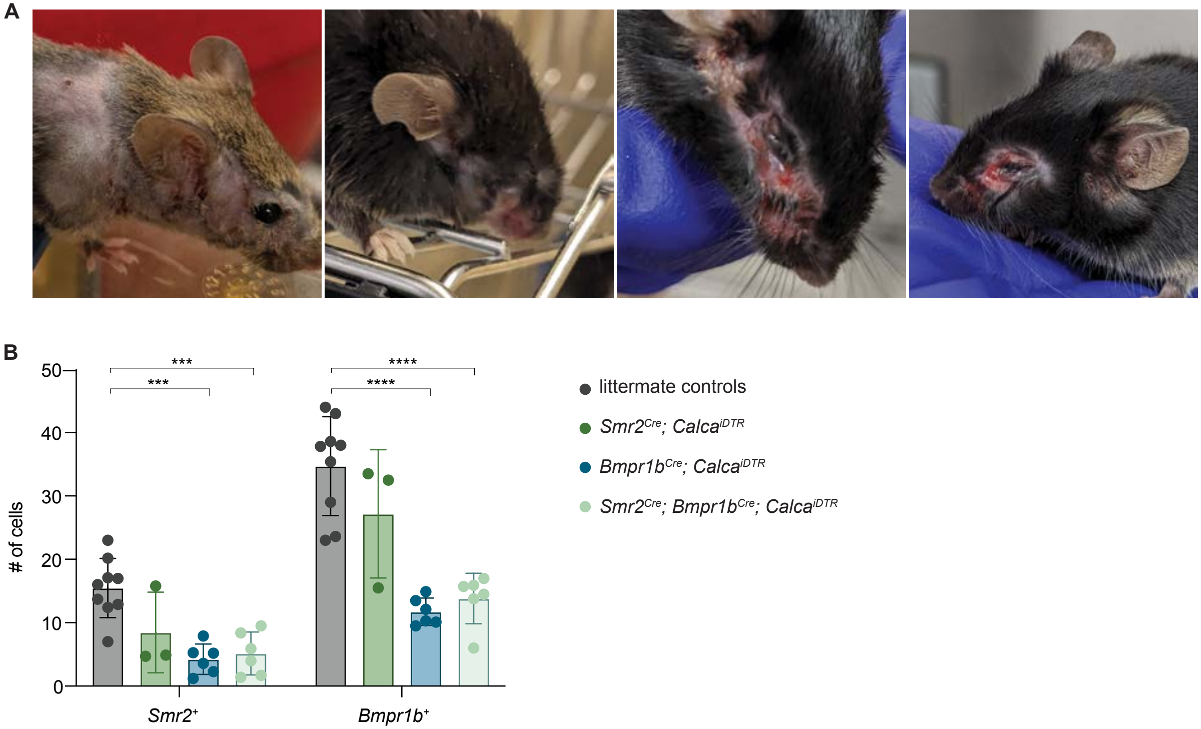
A
B
50
40
30
20
10
0
# of cells
***
***
****
****
Smr2+
Bmpr1b+
littermate controls
Smr2Cre; CalcaiDTR
Bmpr1bCre; CalcaiDTR
Smr2Cre; Bmpr1bCre; CalcaiDTR

Figure S5

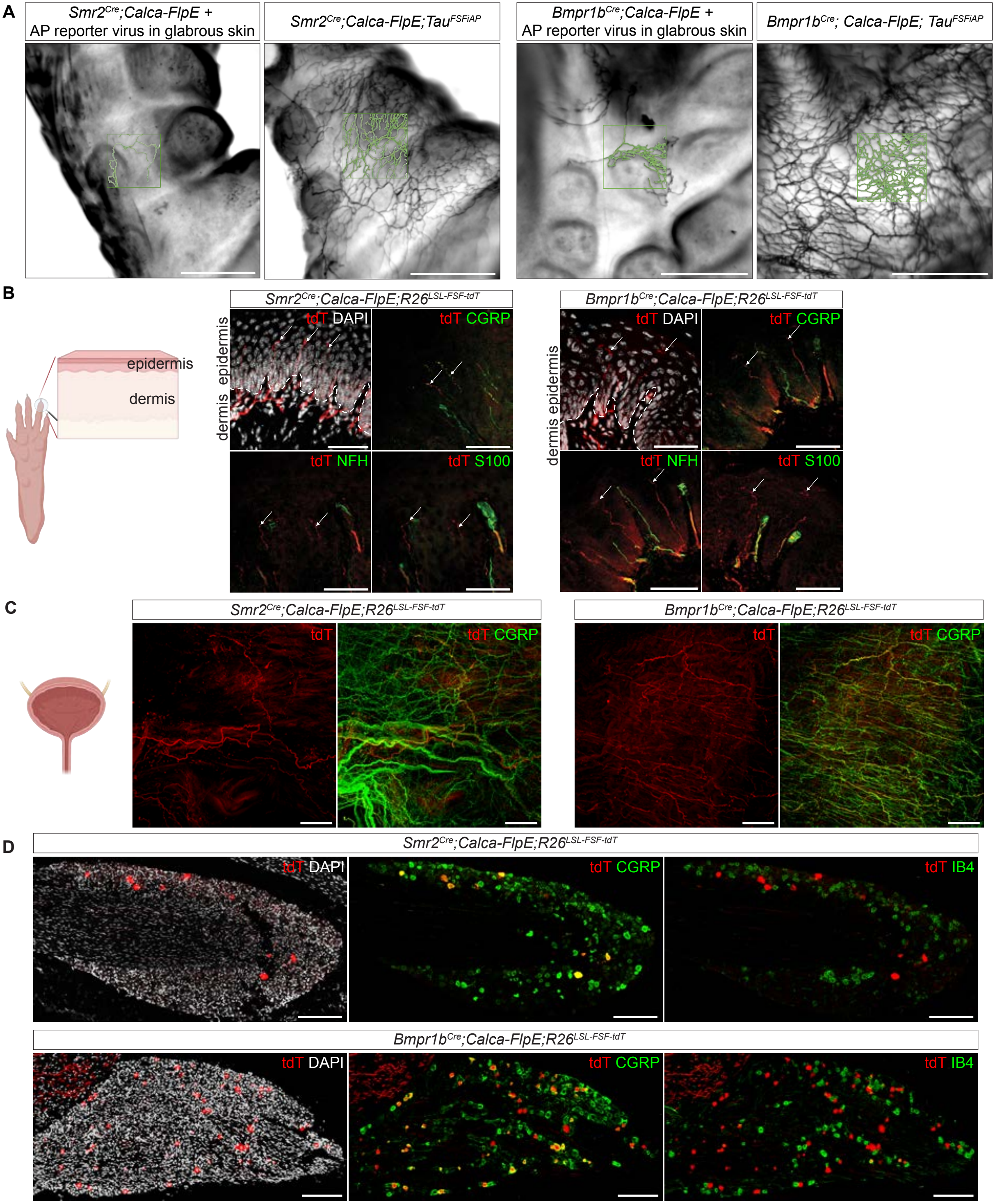

A
Smr2Cre;Calca-FlpE +
AP reporter virus in glabrous skin
Smr2Cre;Calca-FlpE;TauFSFiAP
Bmpr1bCre;Calca-FlpE +
AP reporter virus in glabrous skin
Bmpr1bCre; Calca-FlpE; TauFSFiAP
B
epidermis
dermis
Smr2Cre;Calca-FlpE;R26LSL-FSF-tdT
dermis epidermis
tdT DAPI
tdT CGRP
tdT NFH
tdT S100
Bmpr1bCre;Calca-FlpE;R26LSL-FSF-tdT
dermis epidermis
tdT DAPI
tdT CGRP
tdT NFH
tdT S100
C
Smr2Cre;Calca-FlpE;R26LSL-FSF-tdT
tdT
tdT CGRP
Bmpr1bCre;Calca-FlpE;R26LSL-FSF-tdT
tdT
tdT CGRP
D
Smr2Cre;Calca-FlpE;R26LSL-FSF-tdT
tdT DAPI
tdT CGRP
tdT IB4
Bmpr1bCre;Calca-FlpE;R26LSL-FSF-tdT
tdT DAPI
tdT CGRP
tdT IB4

Figure S6

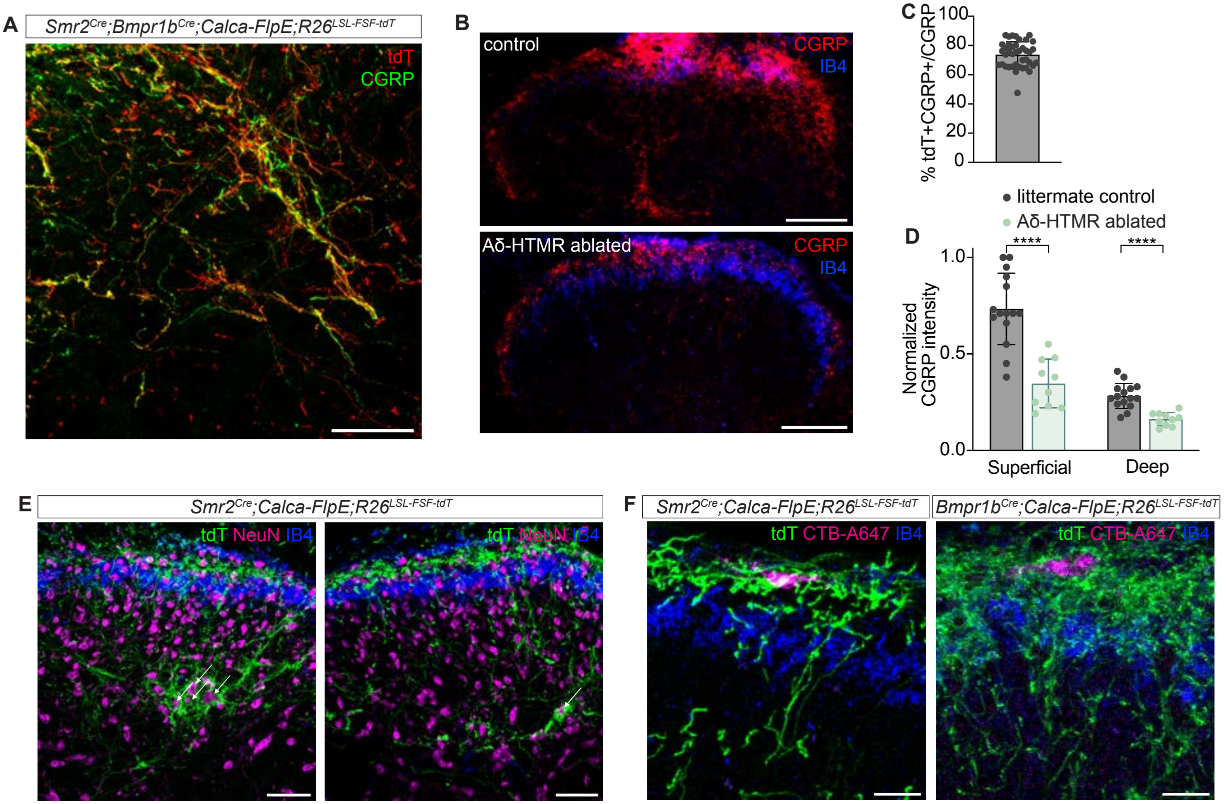

A
Smr2Cre;Bmpr1bCre;Calca-FlpE;R26LSL-FSF-tdT
tdT
CGRP
B
control
CGRP
IB4
Aδ-HTMR ablated
CGRP
IB4
C
% tdT+CGRP+/CGRP
100
80
60
40
20
0
littermate control
Aδ-HTMR ablated
D
Normalized CGRP intensity
1.0
0.5
0.0
****
****
Superficial
Deep
E
Smr2Cre;Calca-FlpE;R26LSL-FSF-tdT
tdT NeuN IB4
tdT NeuN IB4
F
Smr2Cre;Calca-FlpE;R26LSL-FSF-tdT
Bmpr1bCre;Calca-FlpE;R26LSL-FSF-tdT
tdT CTB-A647 IB4
tdT CTB-A647 IB4

Figure S7
